# The toxic effect of R350P mutant desmin in striated muscle of man and mouse

**DOI:** 10.1007/s00401-014-1363-2

**Published:** 2014-11-14

**Authors:** Christoph S. Clemen, Florian Stöckigt, Karl-Heinz Strucksberg, Frederic Chevessier, Lilli Winter, Johanna Schütz, Ralf Bauer, José-Manuel Thorweihe, Daniela Wenzel, Ursula Schlötzer-Schrehardt, Volker Rasche, Pavle Krsmanovic, Hugo A. Katus, Wolfgang Rottbauer, Steffen Just, Oliver J. Müller, Oliver Friedrich, Rainer Meyer, Harald Herrmann, Jan Wilko Schrickel, Rolf Schröder

**Affiliations:** 1 Center for Biochemistry, Institute of Biochemistry I, Medical Faculty, University of Cologne, Joseph-Stelzmann-Str. 52, 50931 Cologne, Germany; 2 Department of Internal Medicine II, University Hospital Bonn, 53105 Bonn, Germany; 3 Institute of Neuropathology, University Hospital Erlangen, Schwabachanlage 6, 91054, Erlangen, Germany; 4 Department of Internal Medicine III, University Hospital Heidelberg, 69120 Heidelberg, Germany; 5 DZHK (German Center for Cardiovascular Research), Partner Site Heidelberg/Mannheim, Heidelberg, Germany; 6 Institute of Physiology I, Life and Brain Center, University of Bonn, 53127 Bonn, Germany; 7 Department of Opthalmology, University Hospital Erlangen, 91054 Erlangen, Germany; 8 Department of Internal Medicine II, University Hospital Ulm, 89081 Ulm, Germany; 9 Core Facility Small Animal Imaging, University of Ulm, 89081 Ulm, Germany; 10 Functional Architecture of the Cell, German Cancer Research Center (DKFZ), 69120 Heidelberg, Germany; 11 Institute of Medical Biotechnology, University of Erlangen, 91052 Erlangen, Germany; 12 Institute of Physiology II, Medical Faculty, University of Bonn, 53115 Bonn, Germany

**Keywords:** Desminopathy, R350P desmin missense mutation, Desmin knock-in mouse, Mouse model, Mutant desmin, Intermediate filament, Extrasarcomeric intermediate filament network, Mechanical vulnerability, Protein aggregation myopathy, Skeletal muscle weakness, Protein aggregation cardiomyopathy, Cardiac arrhythmia, Cardiac conduction defect

## Abstract

Mutations of the human desmin gene on chromosome 2q35 cause autosomal dominant, autosomal recessive and sporadic forms of protein aggregation myopathies and cardiomyopathies. We generated R349P desmin knock-in mice, which harbor the ortholog of the most frequently occurring human desmin missense mutation R350P. These mice develop age-dependent desmin-positive protein aggregation pathology, skeletal muscle weakness, dilated cardiomyopathy, as well as cardiac arrhythmias and conduction defects. For the first time, we report the expression level and subcellular distribution of mutant versus wild-type desmin in our mouse model as well as in skeletal muscle specimens derived from human R350P desminopathies. Furthermore, we demonstrate that the missense-mutant desmin inflicts changes of the subcellular localization and turnover of desmin itself and of direct desmin-binding partners. Our findings unveil a novel principle of pathogenesis, in which not the presence of protein aggregates, but disruption of the extrasarcomeric intermediate filament network leads to increased mechanical vulnerability of muscle fibers. These structural defects elicited at the myofiber level finally impact the entire organ and subsequently cause myopathy and cardiomyopathy.

**Electronic supplementary material:**

The online version of this article (doi:10.1007/s00401-014-1363-2) contains supplementary material, which is available to authorized users.

## Introduction

Desmin is a member of the intermediate filament (IF) protein gene family, which comprises 70 members and represents one of the most highly mutated groups of related genes in the human genome [21]. Mutations in human IF genes have been linked to at least 94 different disease entities ranging from skin, hair and nail diseases (keratins, lamin A/C), eye disorders (keratins, vimentin, phakinin), degenerative disorders of the central and peripheral nervous system (glial fibrillary acidic protein, neurofilament proteins, peripherin, lamin A/C), premature aging syndromes (lamin A/C), lipodystrophy and metabolic syndromes (lamin A/C) to skeletal muscle and cardiac diseases (lamin A/C, desmin) [34, 41].

Desmin, the most abundant IF protein in skeletal, cardiac and smooth muscle cells, is the central component of the filamentous extrasarcomeric cytoskeleton, which links neighboring myofibrils and connects the myofibrillar apparatus with the subsarcolemmal cytoskeleton, myonuclei, mitochondria, intercalated discs as well as myotendinous and neuromuscular junctions. This three-dimensional framework has essential roles in the alignment and anchorage of myofibrils, the resistance of muscle cells against mechanical stress, the positioning of cell organelles, and signaling events [12]. Desmin exhibits a tripartite structure with a central α-helical rod domain flanked by non-α-helical amino-terminal head and carboxy-terminal tail domains. The rod domain consists of two continuous α-helical segments, which are interconnected by a linker (L12) polypeptide sequence. Rod domain coil 1 itself comprises two α-helical subdomains, which are tied by a second linker (L1) sequence [9, 12, 20, 33] (Fig. S1a).

Human desmin, a 470-amino acid protein with a calculated molecular mass of 53.5 kDa, is encoded by a single copy gene (*DES*) on chromosome 2q35 [27]. Since the first description of myopathy- and cardiomyopathy-causing *DES* mutations [16], over 70 mutations have been reported, which spread over the entire *DES* gene, thus affecting the structure and function of the head, rod, and tail domains of the protein [12]. A significant clustering of *DES* mutations is observed in exon 6, which encodes the C-terminal half of the coil 2 domain within the desmin rod (Fig. S1a). The vast majority of genetically proven desminopathies follows an autosomal dominant trait of inheritance. In addition, rare autosomal recessive cases with an earlier and more severe disease manifestation as well as an increasing number of sporadic desminopathies have been described [12].

Human desminopathies are characterized by a marked phenotypic variability with either pure skeletal muscle or cardiac pathology or a combination of both. The progressive skeletal muscle disease may manifest as distal, limb girdle, scapuloperoneal, or generalized myopathy phenotypes. Cardiac disease manifestation comprises true cardiomyopathy, conduction defects, and arrhythmias [12]. Desminopathies are morphologically characterized by sarcoplasmic and subsarcolemmal desmin-positive protein aggregates and degenerative changes of the myofibrillar apparatus. They are the classical protagonists of the expanding group of myofibrillar myopathies (MFMs), a numerically significant subgroup of hereditary and sporadic protein aggregate myopathies with marked clinical and genetic heterogeneity due to mutations of the desmin, αB-crystallin, BAG-3, FHL1, filamin-C, myotilin, plectin, and ZASP genes [37].

We previously described the clinical, myopathological, and molecular consequences of the human heterozygous R350P *DES* mutation in several German families [3, 46]. This missense mutation residing in exon 6 (Fig. S1a) is the most frequently encountered gene defect causing desminopathies and leads to a single amino acid exchange from arginine to proline at position 350, which represents a b position in the heptad pattern characteristic for coiled coil forming α-helices. Actually, arginine 350 is part of the sole undeca-repeat in the center of coil 2 that harbors the “stutter”. Here, both helices of the coiled coil exhibit a short-unwound region as demonstrated for the corresponding, nearly identical domain of the vimentin dimer [39]. In transfection studies the R350P desmin mutant was not capable to form a de novo desmin network in IF-free cells, disrupted the pre-existing, endogenous vimentin IF network in 3T3 cells, and led to the formation of cytoplasmic protein aggregates. Moreover, R350P desmin showed a highly abnormal pattern in in vitro desmin filament assembly experiments. R350P desmin aborted the normal filament assembly already at an early stage and led to pathological protein aggregation. Already the presence of 25 % of the mutant desmin effectively aborted the normal polymerization process of desmin IFs [3].

Studies on the molecular pathogenesis of human desminopathies are generally hampered by the fact that muscle biopsies from affected patients reflect only late stages of the disease process, are only available in small amounts, and biopsy material from pre-clinical, early and intermediate disease stages is not accessible [12]. Thus, we generated a R349P desmin knock-in mouse model for human desminopathies. Since murine desmin, compared to human desmin, lacks a serine at position 82 (Fig. S1a), murine R349 is the ortholog of human R350 (both proteins further differ in 11 conservative amino acid changes; sequence identity is 97 %). Here, we report the clinical, electrophysiological, hemodynamic, radiological, myopathological, biomechanical, and molecular findings in heterozygous (HET) and homozygous (HOM) R349P desmin knock-in mice as compared to wild-type (WT) littermates. Our knock-in mouse strain represents the first physiological animal model for autosomal dominant and recessive human desminopathies, as the expression of the mutant desmin is controlled by the endogenous gene regulation sites.

## Materials and methods

For complete and detailed information on the animal model, all methods, materials, and devices used in this study please refer to the Supplemental Methods. Data analyses and statistical evaluations were performed using Excel 2010 (Microsoft); the Kruskal–Wallis one-way analysis of variance, Mann–Whitney *U* (Wilcoxon rank-sum), Fisher’s exact, Chi-squared, and 1-way ANOVA with Bonferroni subgroup statistical tests were done using the Excel add-in “Real Statistics Resource Pack” version 3.1.2 by Charles Zaiontz available at http://www.real-statistics.com/; the Lord test was manually calculated according to [22]. When the Mann–Whitney *U* test was used for post hoc analyses, it was used without further correction of the level of significance, as only two samples were compared to a single control. The number of independent experiments, mean values, standard errors, type of statistical test, and significance levels are indicated in the “Results” section or Figure legends. Final assembly and preparation of all figures for publication was done using Corel Draw Graphics Suite X4.

### Generation and genotyping of R349P desmin knock-in mice

The R349P desmin knock-in mouse model B6J.129Sv-*Des* ^tm1(R349P)Cscl&Rfsr^ was generated according to our specifications (CSC, RS) by genOway, Lyon, France. Routine genotyping was performed by PCR. In addition, mice were genotyped at random by Southern blotting, and the presence of the R349P desmin point mutation was verified by sequencing. Mice were housed in isolated ventilated cages (IVC) under specific and opportunistic pathogen-free (SOPF) conditions at a standard environment with free access to water and food. Health monitoring was done as recommended by the Federation of European Laboratory Animal Science Associations (FELASA). Mice were handled in accordance with the German Animal Welfare Act (Tierschutzgesetz) as well as the German Regulation for the protection of animals used for experimental purposes or other scientific purposes (Tierschutz-Versuchstierverordnung), and the investigations were approved by the responsible governmental animal care and use office (North Rhine-Westphalia State Agency for Nature, Environment and Consumer Protection (LANUV), Recklinghausen, Germany; reference number 8.87-50.10.31.09.045).

### Investigation of striated muscle function

Grip strength was measured using a BIO-GS3+ grip strength meter (Bioseb, Vitrolles, France). For the wire hanging test mice were placed on a wire cage lid, which was slowly turned upside down. Electrostimulation of explanted soleus muscles for determination of twitch and tetanic force recordings were performed as described previously (supplemental experimental procedures in [1]). For passive-stretch experiments, small fiber bundles of five single fibers were dissected from soleus muscles and transferred to an automated force transducer system with step-wise length increases according to [44].

### Cardiac phenotyping

Functional cardiac magnetic resonance imaging (CMR) of the left ventricle was performed using a 4-element cardiac phased-array coil on a dedicated 11.7 T small animal MRI system (BioSpec 117/16, Bruker, Ettlingen, Germany), applying a self-gated imaging technique (IntraGate, Bruker, Ettlingen, Germany [19]). For echocardiography, a Sonos 5500 (Philips, Eindhoven, The Netherlands) with a S12 transducer (12 MHz) was used, and the performing person was blinded during procedure as previously described [36]. Pressure volume loop recordings were made in closed-chest, spontaneously breathing mice using a 1.2-French catheter (Model FT111B, SciSense Inc., London, ON, Canada) inserted into the left ventricle of the mice to simultaneously measure pressure and volumes as previously described [5, 36].

### Surface, long-term, and intracardiac electrophysiological investigations

A surface 6-lead electrocardiogram was continuously monitored and analyzed under stable conditions [2]. For long-term electrocardiography analysis in conscious animals, telemetry devices (Modell EA-F20; DataSciences International, St. Paul, MN, USA) were implanted according to [14], and the leads were directed ventrally and fixed to the pectoral muscle in an Eindhoven II position. Recordings were performed 10 days after recovery from surgical instrumentation. All baseline recordings were performed in conscious animals for 24 h in a constant environment. At the end of the baseline recording a physical stress test, i.e., 10 min swimming exercise, was performed under continuation of electrocardiogram recording as described before [23]. In vivo transvenous electrophysiological investigations were performed using a 2-French octapolar mouse electrophysiological catheter (eight 0.5 mm circular electrodes, electrode-pair spacing 0.5 mm; Ciber Mouse, NuMed Inc., NY, USA) positioned in the right cardiac cavities on atrial and ventricular level. Intracardiac electrograms and transvenous atrial and ventricular stimulation maneuvers were registered and recorded as previously described [35].

### Human skeletal muscle biopsy material

Tissue samples of skeletal muscle derived from a diagnostic muscle biopsy of a patient from a previously reported family with a heterozygous R350P desmin mutation [46] were obtained from the Friedrich-Baur-Institute, Munich, Germany.

### Preparation of striated muscle sections, immunohistochemistry, immunofluorescence stains, and ultrastructural analysis

Skeletal and cardiac muscle specimens were collected, immediately frozen in liquid nitrogen-cooled isopentane, sectioned, further prepared, and used for standard histology and immunofluorescence stains; images were acquired using a LSM780 fluorescence laser scanning microscope (Carl Zeiss GmbH, Oberkochen, Germany) and a Leica TCS SP5/AOBS/tandem scanning system (Leica Microsystems GmbH, Wetzlar, Germany). For quantitation of myocardial fibrosis, sections of paraffin embedded myocardial whole heart samples were stained with Sirius red, and fibrosis was defined as the percent area of extracellular Sirius red staining. Murine cardiomyocytes were isolated according to [42], and immunofluorescence analyses were performed after attachment of the cells to laminin-coated microscope slides. For transmission electron microscopy, soleus muscle specimens were prepared following standard protocols and examined with a LEO906E transmission electron microscope (Carl Zeiss GmbH, Oberkochen, Germany).

### Real-time polymerase chain reactions

Analyses of the mRNA expression levels of brain natriuretic peptide (BNP), desmin, and desmin-binding partners were done using cDNA derived from striated muscle of the R349P desmin knock-in mice. Quantitative real-time PCR was performed on an ABI PRISM 7000 (Applied Biosystems, Foster City, USA), an Opticon II instrument (MJ Research Inc., St. Bruno, Quebec, Canada), and a CFX Connect Real-Time PCR Detection System (Bio-Rad Laboratories, Hercules, CA, USA). GAPDH, AIP1, and HPRT genes were used as reference genes, appropriate quality controls were performed, and data were analyzed using the delta–delta-*C*_q_-Method (ΔΔ*C*_q_). Expression ratios are displayed as fold change in relation to the wild-type control samples.

### Determination of wild-type and R350P/R349P mutant desmin mRNA levels

Complementary DNA derived from striated muscle of a R350P desminopathy patient and HET R349P desmin knock-in mice was used for PCR, and PCR products were either directly subjected to restriction digestion or first cloned into pGEM-Teasy (Promega Corporation, Madison, WI, USA) for transformation into *E. coli*, growth of colonies, use for colony PCR, and then subjected to restriction digestion. As the desmin point mutation destroys an endogenous AciI restriction site in human and inserts a novel AvaI restriction site in mouse, the amounts of wild-type and mutant desmin mRNA can be determined.

### Desmin antibodies

Two desmin antibodies were newly generated for the purpose of this study. Rabbits were immunized with hepta-peptides surrounding amino acid residue 350/349 of desmin (wild-type peptide C-MRQMREL, mutant peptide C-MRQMPEL; PSL Peptide Specialty Laboratories GmbH, Heidelberg, Germany) and affinity purified. Wild-type R350/R349 desmin specifically was detected by rabbit pAb HD2 (1:1,000 in TBS-T for Western blotting), and R350P/R349P mutant desmin by rabbit pAb HD350P (1:1,000 in TBS-T for Western blotting, 1:200 in PBS for immunofluorescence).

Both wild-type and R350P/R349P mutant desmin were detected by three commercially available “pan-desmin” antibodies (D1033, Sigma-Aldrich (St. Louis, MO, USA), mouse mAb, 1:400 in TBS-T for Western blotting; D33, Dako (Glostrup, Denmark), mouse mAb, 1:400 in PBS-T for Western blotting, 1:50 or 1:100 in PBS for immunofluorescence; 10570, Progen Biotechnik GmbH (Heidelberg, Germany), rabbit pAb, 1:100 in PBS for immunofluorescence). See the Supplemental Methods for information on other antibodies.

### Gel electrophoresis techniques, in vitro dephosphorylation, and mass spectrometry

For reproducible immunoblotting extraction of proteins from striated muscle tissue was done according to [10]. For protein quantitation, a fluorometric dye (ProStain, Active Motif, Carlsbad, CA, USA) was used. 2D-SDS-PAGE was performed according to [11], mass spectrometry according to [13]. In vitro dephosphorylation assays were done as described in [48].

### Expression of recombinant desmin in *E. coli* and insect cells

Recombinant human wild-type and R350P mutant desmin cloned into pTriEx1.1-Neo, which contains a bacterial T7, a mammalian CMV as well as an insect cells baculovirus p10 promoter, were expressed in *E. Coli* BL21(DE3)pLysS cells [40] and Sf9 insect cells.

### Cycloheximide assay

For effective blockade of protein synthesis mice received daily s. c. injections at their neck of 60 mg/kg cycloheximide, typically 180 µl of a 10 mg/ml solution in PBS, for 4 days (d1–d4); first injections (d0) were with PBS only. Control mice daily received only PBS. Mice were killed by cervical dislocation, and tissues were dissected and snap-frozen in liquid nitrogen for further analyses. The administered dose of cycloheximide was derived from [15, 28, 29, 32, 38, 43] and ChemIDplus at http://chem.sis.nlm.nih.gov/chemidplus/ (LD50 mouse s.c.: 160 mg/kg).

## Results

### R349P desmin knock-in mice: a model for human R350P desminopathies

Heterozygous R349P desmin knock-in mice were generated using a gene targeting strategy, which replaced the triplet “AGG” (human “CGG”) encoding arginine by “CCC” (human “CCG”) encoding proline in exon 6. After homologous recombination and removal of the neomycin selection cassette by *Cre* recombinase, the original gene structure is preserved and only a single LoxP site remains in intron 6. The correct targeting event was confirmed by Southern blotting, PCR genotyping, and sequencing of genomic DNA (Fig. S1b–e). Crossbreeding of HET mice resulted in the generation of HOM R349P mice. Though homozygous R350P desmin patients have not been described yet, our HOM mice depict essential aspects of rare autosomal recessive desminopathies due to missense, small in-frame deletion, and compound heterozygous mutations [12]. Both HET and HOM desmin R349P knock-in mice are viable and fertile, showed no overt behavioral abnormalities, and the assessment of body weight, heart weight, heart-to-body weight, heart weight-to-tibia length ratios, and serum creatine kinase levels showed no statistically significant differences between the genotypes (data not shown).

### Expression and decay of wild-type versus point-mutated desmin

**Fig. 1:**
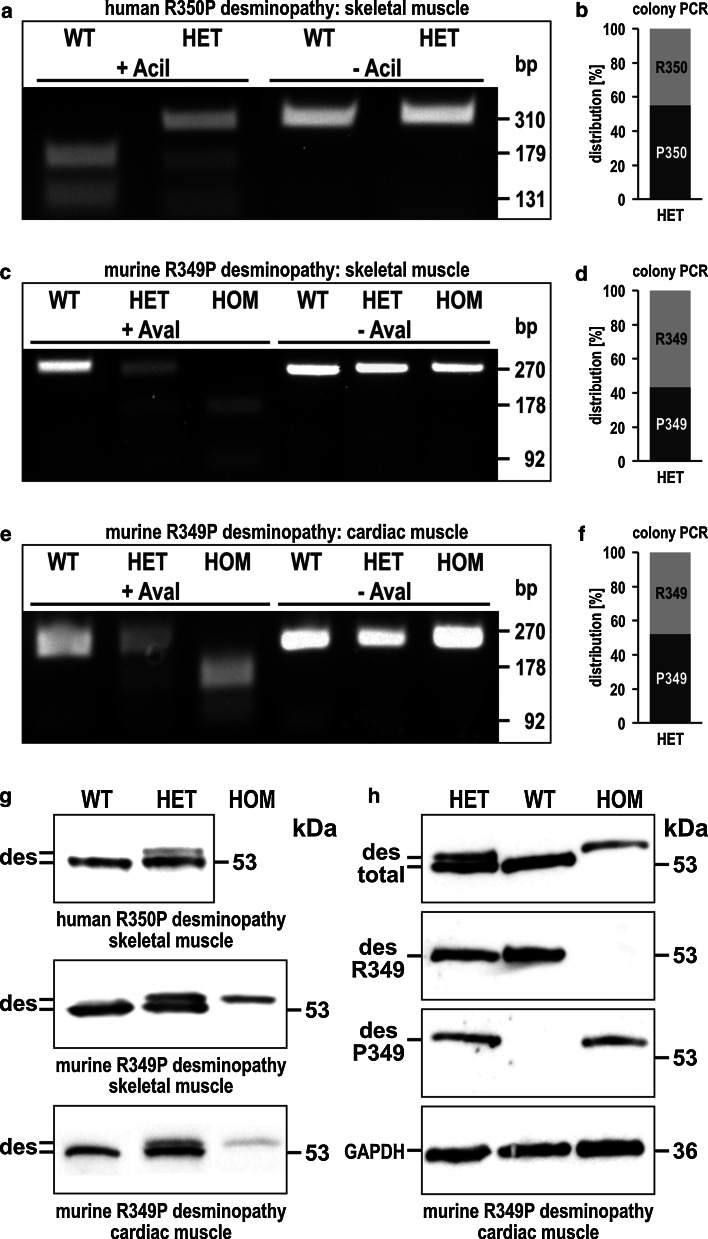
Expression levels of wild-type and mutant desmin mRNA and protein in human and murine striated muscle tissue. **a** To address the expression level of wild-type and R350P mutant desmin mRNA in skeletal muscle tissue from a desminopathy patient, we performed RT-PCRs in conjunction with AciI restriction digestion. Since the presence of the R350P missense mutation destroys an endogenous AciI restriction site, the 310 bp RT-PCR product, a mixture derived from wild-type and mutant mRNA, is incompletely digested into the 179 and 131 bp fragments in the desminopathy patient. **b** To quantitate the levels of wild-type and mutant desmin mRNA, the 310 bp mixed product was cloned into the pGEM-Teasy vector, transformed into *E. coli*, and single colonies were used for a colony PCR. AciI restriction digestions of these PCR products resulted in either 310 bp or the 179 and 131 bp fragments, and were used to determine the relative amounts of wild-type and mutant desmin mRNA in the heterozygous R350P desminopathy patient. **c**–**f** Analogous experiments were performed in skeletal and cardiac muscle tissue derived from R349P desmin knock-in mice. Here, the presence of the R349P missense mutation creates a novel AvaI restriction site. Note, that the relative amounts of wild-type and mutant desmin mRNA in skeletal and cardiac muscle from HET mice are within a similar range as the one observed in human R350P desminopathy skeletal muscle. **a**, **c** For illustration, *lines* from the same agarose gel were digitally re-arranged. **g** The presence of the R350P and R349P mutation in man and mouse, respectively, alters the migration pattern of the desmin molecule in SDS-PAGE. Since the mutant displayed a slower migration pattern, desmin immunoblotting allows quantitation of wild-type and mutant desmin on the protein level. *Upper panel*, two distinct desmin bands are visible in skeletal muscle tissue from a desminopathy patient. The amount of mutant desmin (upper band) is significantly reduced as compared to the amount of wild-type desmin. *Middle* and *lower panels*, corresponding immunoblots using skeletal and cardiac muscle tissue from WT, HET, and HOM mice. In WT and HOM animals, only single bands of desmin were detected, whereas HET mice displayed the double-band pattern as observed in the human situation. Note the markedly reduced levels of R349P mutant desmin protein in HOM mice. Protein concentrations were determined using the “ProStain” fluorometric dye (Active Motif) and equal amounts of proteins from WT, HET, and HOM tissue lysates were loaded. **h** Immunoblotting of wild-type versus R349P mutant desmin in cardiac muscle tissue from knock-in mice using a set of three different desmin-specific antibodies. *Uppermost lane* detection of wild-type and mutant desmin using a commercially available mouse monoclonal desmin antibody. *Second panel* we generated a rabbit polyclonal antibody that specifically detects the wild-type (R349) desmin in WT and HET mice, but not the mutant. *Third panel* exclusive detection of the mutant desmin (P349) in HET and HOM mice by a second newly generated rabbit polyclonal antibody. *Lowermost panel* GAPDH for loading control

A direct comparison of the human and murine desmin-related pathology is a central aspect of the present study. To determine the expression levels of wild-type and R350P mutant desmin mRNAs in humans, we performed RT-PCRs from a diagnostic skeletal muscle biopsy of a German patient with a heterozygous R350P desmin mutation (Fig. 1a). This analysis showed that 55 % of total desmin mRNA is derived from the mutant allele (Fig. 1b). Analogous experiments using skeletal and cardiac muscle tissue from HET R349P desmin knock-in mice revealed 43 and 55 % mutant desmin mRNA, respectively (Fig. 1c–f).

In contrast to our initial immunoblotting which demonstrated a single desmin band at 53 kDa [3], our current analysis using high-resolution SDS-PAGE in conjunction with desmin immunoblotting revealed that the human R350P and the murine R349P mutant desmin proteins migrate slower than wild-type desmin (Fig. 1g). In the desminopathy patient, approximately 20 % of total desmin protein is the mutant variant. In HET mice, the relative quantity of mutant desmin was within a range of 20–50 % in both skeletal and cardiac muscle tissue. Similar amounts of mutant desmin were also observed in Coomassie brilliant blue stained 2D-gels of skeletal and cardiac muscle tissue lysates (data not shown). In HOM mice, only mutant desmin with markedly reduced signal intensity was detected by immunoblotting. Here, the amount of mutant desmin was in a range from hardly detectable up to 30 % as compared to the signal intensity wild-type desmin.

To further address the aberrant migration pattern of mutant desmin with respect to putative differential post-translational modifications, we expressed recombinant human desmin in *E.* *coli*, Sf9 insect and HEK293 mammalian cells. Mutant desmin expressed in these different systems, which are, respectively, limited, partially, and fully capable of mammalian post-translational modifications, displayed identical migration patterns in SDS-PAGE and urea-PAGE (Fig. S2a). Moreover, no decrease in the higher apparent molecular mass of mutant desmin could be observed in in vitro dephosphorylation assays using protein extracts from human as well as murine skeletal muscle tissue (Fig. S2b). Thus, the aberrant migration pattern of mutant desmin must be due to structural changes induced by the replacement of the arginine by a proline side chain in the desmin rod domain coil 2.

**Fig. 2:**
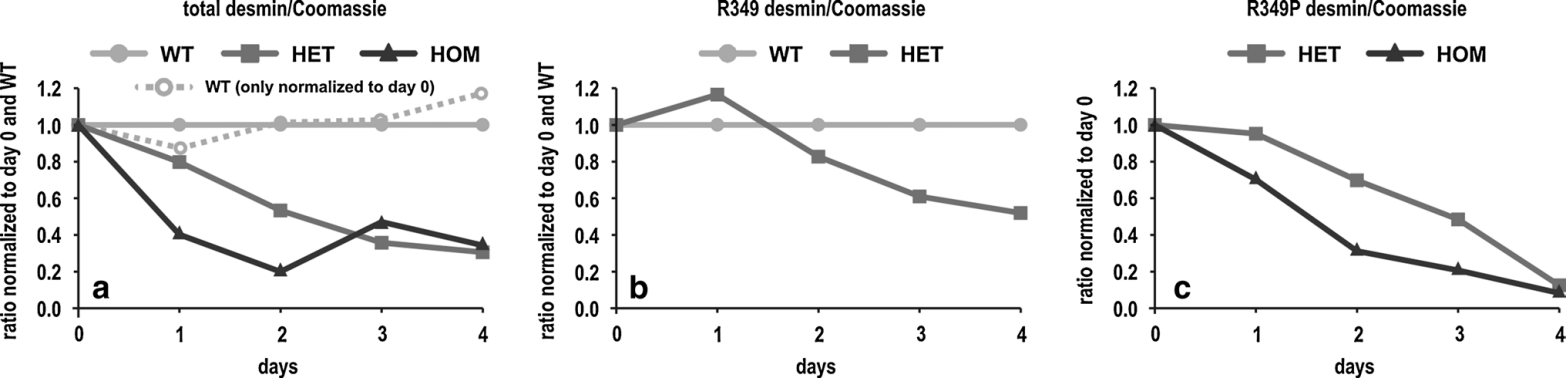
Enhanced desmin turnover in R349P desmin knock-in mice. **a**–**c** Wild-type and R349P mutant desmin protein levels in skeletal muscle tissue of mice, in which protein synthesis was completely inhibited by cycloheximide treatment over a time span of 4 days. The amounts of wild-type and R349P mutant desmin were determined by immunoblotting using the pan-desmin, R349 wild-type-, and P349 mutant-specific antibodies. Graphs were generated from densitometry analyses of single, representative experiments, and the obtained desmin intensity values were normalized to both the total protein content as determined by Coomassie Brilliant Blue stained SDS-PAGE gels and the desmin densitometry values at day 0. In **a** and **b** the densitometry values of HET and HOM mice were additionally normalized to the ones of WT animals. **a** Determination of the total desmin amount using the pan-desmin antibody revealed that the decline of desmin in HET and HOM mice is accelerated as compared to WT animals. The second, *dotted* *WT curve* illustrates the amount of desmin in WT mice only normalized to day 0. **b** Determination of the amount of wild-type desmin (R349) showed that it also declined faster in HET mice. **c** Determination of the amount of R349P mutant desmin in HET and HOM animals. Here, the mutant desmin in HOM mice declined more rapidly than in HET mice, which express both wild-type and mutant desmin

Since the commercially available desmin antibody detects both the mutant and the wild-type desmin, we generated rabbit polyclonal antibodies that specifically detect either the mutant or the wild-type desmin (Fig. 1h). Using these antibodies, we investigated the wild-type and R349P mutant desmin decay in skeletal muscle tissue of mice, in which protein synthesis was completely inhibited by cycloheximide treatment (Fig. 2a–c). These studies demonstrated that the decay of desmin in HET and HOM mice was accelerated when compared to WT animals. Moreover, not only the mutant, but also the wild-type desmin content showed a faster decay in HET mice, and mutant desmin disappeared more rapidly in HOM than in HET mice.

### Structural pathology of skeletal and cardiac muscle in R349P desmin knock-in mice

**Fig. 3:**
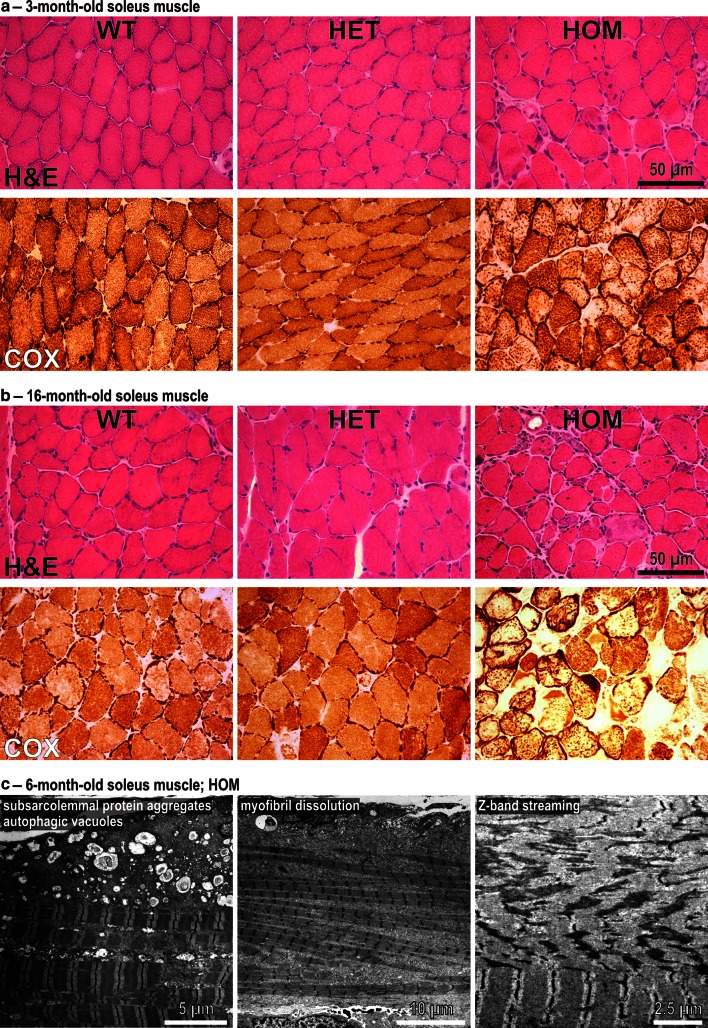
Age-related skeletal muscle pathology and ultrastructural analysis of soleus muscle in R349P desmin knock-in mice. **a** Hematoxylin and eosin (H&E) and cytochrome C oxidase (COX) stains of transverse cryosections from 3-month-old animals. While HET mice showed no overt pathology, HOM mice displayed moderate degenerative muscle changes with increased connective tissue, pathological variation of fiber diameters, and centralization of myonuclei. In addition, COX stains revealed multiple fibers with areas of diminished enzyme staining. **b** Marked, age-related progression of dystrophic alterations in 16-month-old HOM mice. In addition to a further increase in connective tissue, degenerating fibers became apparent. Compared to 3-month-old animals, the aged mice also showed a marked accentuation of the mitochondrial pathology as revealed by COX staining. Note the presence of multiple fibers with strongly diminished sarcoplasmic and/or increased subsarcolemmal enrichment of enzyme staining. **c** In addition to massive subsarcolemmal protein aggregate pathology and autophagic vacuoles, various degenerating myofibrils, and marked Z-band streaming as additional signs of myofibril degeneration are depicted

Our myopathological analysis revealed clear evidence of skeletal muscle myopathy in HOM mice, in which we observed an age-related increase in the extent of degenerative muscle changes consisting of increased endomysial connective tissue, rounding of muscle fibers, pathological fiber sizes variations, centralization of myonuclei, and degenerating muscle fibers in soleus muscle tissue. These age-related changes were accompanied by a corresponding aggravation of mitochondrial abnormalities comprising multiple muscle fibers with focal changes of cytochrome C oxidase (COX) (Fig. 3a, b) and succinate dehydrogenase (SDH) (data not shown) enzyme activities. Similar myopathic alterations were detected in the diaphragm of HOM mice, while no such changes could be detected in gastrocnemius and quadriceps femoris muscles (data not shown). At the ultrastructural level, soleus muscle from HOM mice showed massive subsarcolemmal protein aggregate pathology, autophagic vacuoles, Z-band streaming, and lysis of myofibrils (Fig. 3c). In line with the highly abnormal COX staining, electron microscopy further revealed focal accumulation or depletion of mitochondria as well as giant mitochondria. The analysis of skeletal muscle tissue from 3- and 16-month-old HET R349P mice showed no overt myopathic alterations indicating that the observed expression level of R349P mutant desmin is not sufficient to induce progressive skeletal muscle damage in these mice. In contrast, the morphological analysis of cardiac muscle tissue revealed pathological alterations consistent with a cardiomyopathy in both genotypes. Here, H&E and Sirius red stains revealed increased connective tissue in HET and, more prominent, in HOM mice (Fig. S3).

### Desmin protein aggregation pathology in skeletal and cardiac muscle

**Fig. 4:**
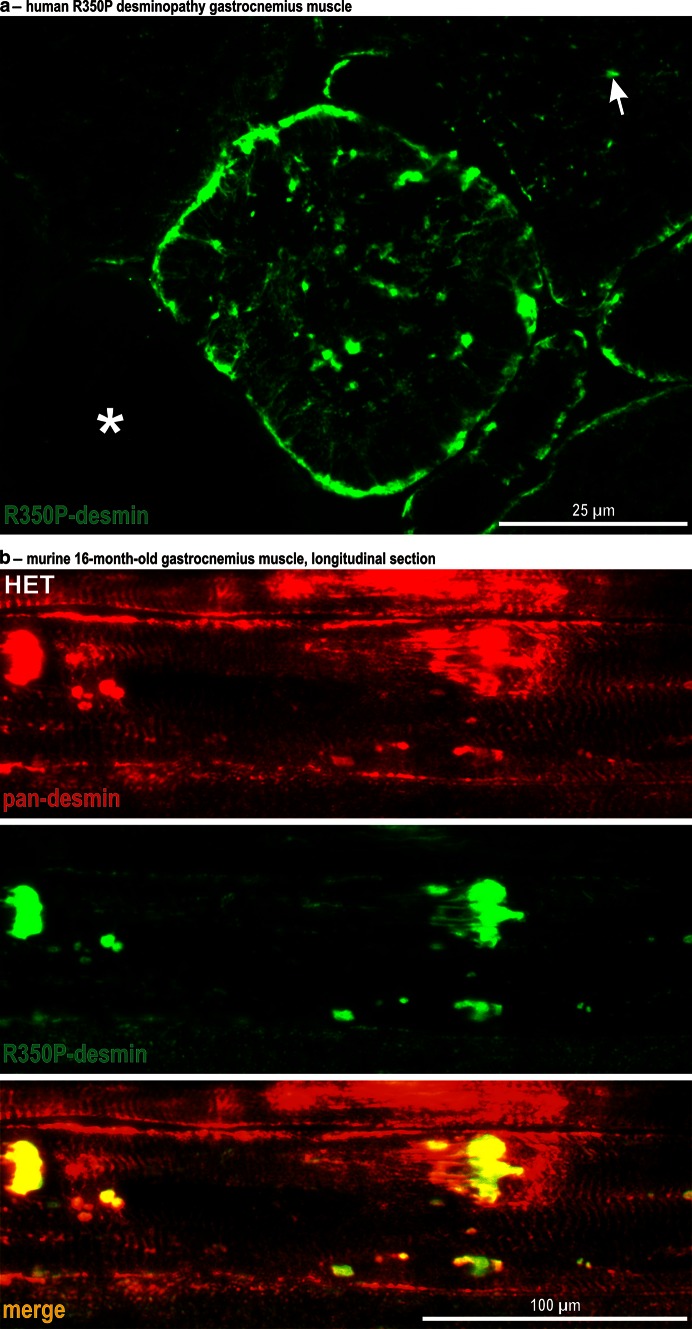
Subcellular distribution of wild-type and mutant desmin in human and murine skeletal muscle tissue. **a** Analysis of human R350P desminopathy skeletal muscle tissue using the R350P desmin mutation-specific antibody. High magnification of a single muscle fiber containing massive subsarcolemmal and multiple sarcoplasmic protein aggregates. Neighboring fibers showed less alterations (*arrow*) or almost no expression of the mutant desmin (*asterisk*). **b** Pan-desmin and R349P desmin stains in longitudinal sections of murine gastrocnemius muscle derived from HET mice. Note, that the regular cross-striated desmin pattern (pan-desmin) is mainly preserved. In contrast, the R350P desmin is only present in pathological protein aggregates (*yellow* in the merged image)

Using the newly generated mutant desmin-specific antibody, we were able to reveal the subcellular distribution of the mutant desmin in human and murine muscle tissue [Fig. 4 (for details); Fig S4 (for overview)]. Analysis of cross-sections from a diagnostic muscle biopsy specimen of a R350P desminopathy patient revealed that the R350P-specific antibody only labeled a subset of fibers in which the mutant desmin was primarily localized in the subsarcolemmal region and, to a lesser extent, in small sarcoplasmic aggregates (Fig. S4b). At higher magnification, a mixture of fibers with gross alterations of the sarcoplasmic desmin network and protein aggregates, and fibers with virtually no R350P desmin expression could be visualized (Fig. 4a). Corresponding desmin immunostains were performed in soleus muscle from 3- and 16-month-old R349P desmin knock-in mice (Fig. S4c, d). Using the pan-desmin antibody, muscle tissue from young HOM animals already displayed a highly abnormal staining pattern with fibers containing multiple subsarcolemmal and sarcoplasmic desmin-positive protein aggregates as well as multiple fibers with virtually no sarcoplasmic desmin staining. In contrast, pan-desmin stains in HET R349P mice showed no obvious changes in comparison to age-matched wild-type controls. Using the mutant-specific desmin antibody, however, the presence and an age-dependent increase of R349P desmin protein aggregation pathology could be visualized in soleus muscle of HET and HOM mice (Fig. S4c, d). Furthermore, comparison of the protein aggregation pathology in different muscle groups revealed that the level of mutant desmin was much more abundant in diseased soleus muscle than in gastrocnemius and quadriceps femoris muscles, which showed no myopathic alterations. Analysis of longitudinal sections of gastrocnemius muscle derived from HET animals demonstrated that the mutant desmin is not incorporated into the cross-striated wild-type desmin cytoskeleton, but segregated into desmin-positive protein aggregates (Fig. 4b; Fig. S4e).

The analysis of cardiac tissue derived from HOM R349P mice showed a highly abnormal desmin-staining pattern in which the mutant desmin is enriched at the level of intercalated discs as well as in small dotted protein aggregates in the sarcoplasm of individual cardiomyocytes (Fig. S5a). It is noteworthy that these small protein aggregates often displayed a seemingly regular dotted pattern in homozygous tissue and isolated cardiomyocytes. Though the normal cross-striated desmin-staining pattern, which is seen in HET and WT animals, was completely abolished, the latter finding might indicate that the R349P mutant desmin can still associate to the level of the Z-discs, but itself is incapable of forming a filamentous three-dimensional desmin cytoskeleton (Fig. S5b).

### Mutant desmin changes the subcellular localization and turnover of its direct, extrasarcomeric binding partners

**Fig. 5:**
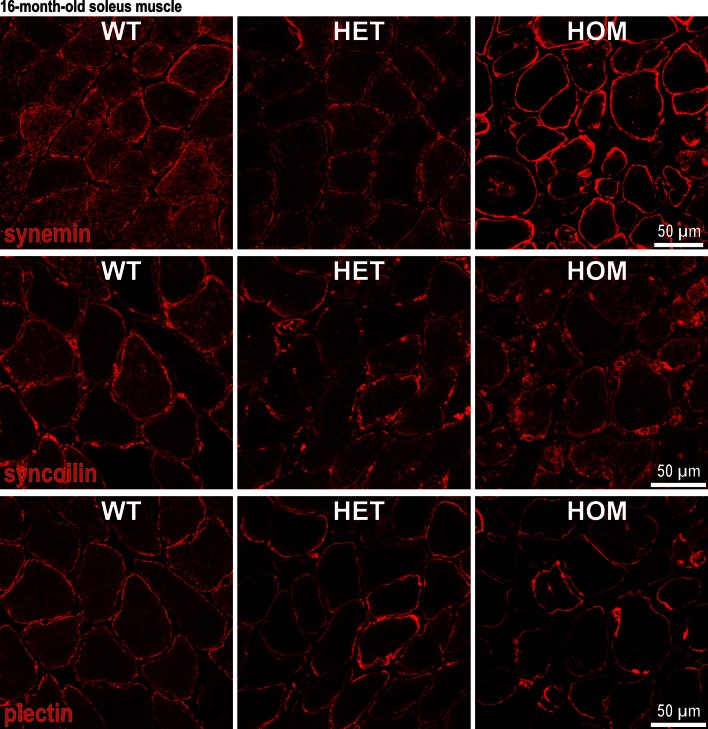
Altered subcellular distribution of direct desmin-binding partners in skeletal muscle tissue of R349P desmin knock-in mice. Synemin, syncoilin, and plectin are well-established direct desmin interaction partners, which together form the extrasarcomeric cytoskeleton in striated muscle cells. All three proteins displayed altered subcellular distributions with accumulation in the subsarcolemmal region and diminished sarcoplasmic staining of muscle fibers in HOM mice. Though to a lesser extent, these changes were also visible in HET mice

The extrasarcomeric cytoskeleton in mature striated muscle cells is primarily composed of desmin, the type IV IF proteins synemin and syncoilin, as well as the multifunctional cytolinker plectin. Synemin, syncoilin, and plectin are well-established direct desmin-binding partners and exert essential roles in the anchorage of pre-formed desmin filaments to the sarcolemma, the periphery of myofibrillar Z-discs, and neuromuscular and myotendinous junctions [12]. In skeletal muscle specimens from desminopathy patients, synemin (Fig. S6), syncoilin (Fig. 7 in [12]), and plectin (Fig. 2 in [3]) co-localized with desmin in subsarcolemmal and sarcoplasmic protein aggregates. In our mouse model, we were able to demonstrate that the expression of R349P mutant desmin severely affected the subcellular distribution of desmin-binding partners of the extrasarcomeric cytoskeleton (Fig. 5). In HOM mice, synemin and plectin were found to be enriched in the subsarcolemmal region and in protein aggregates, while their sarcoplasmic staining signal was markedly reduced. In HET animals, a less strikingly reduced sarcoplasmic staining of synemin and plectin as well as an enhanced subsarcolemmal staining of plectin was visible. Vimentin immunofluorescence imaging showed no evidence of vimentin expression in the diseased skeletal muscle fibers (data not shown).

**Fig. 6:**
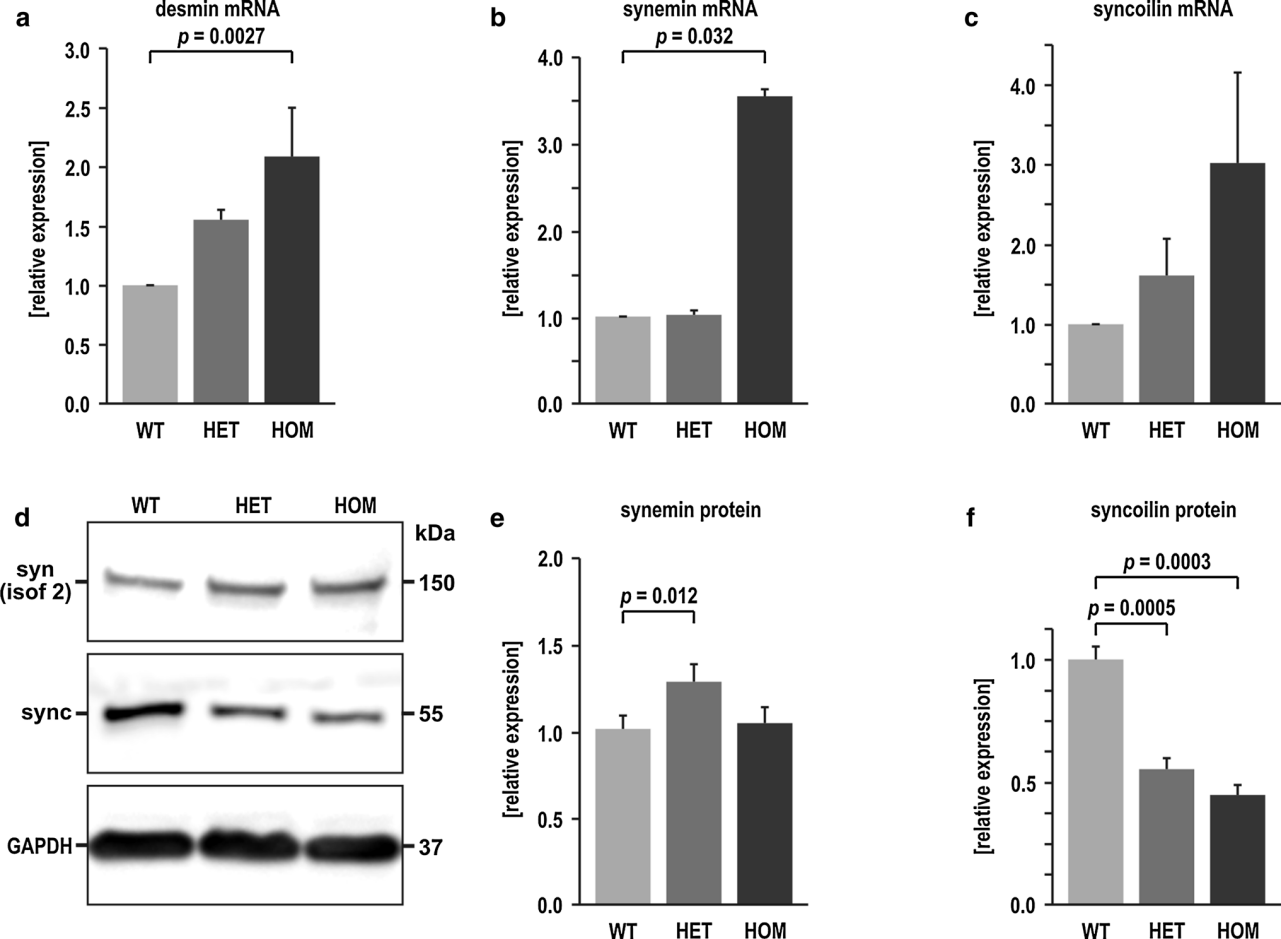
Aberrant intermediate filament expression in R349P desmin knock-in mice. **a**–**c** Quantitative real-time RT-PCR analyses of desmin, synemin, and syncoilin mRNA expression in soleus muscle tissue derived from WT, HET, and HOM mice. Though desmin protein levels were markedly reduced in HOM mice, a significant up-regulation of desmin mRNA is seen in this genotype indicating a highly increased protein turnover. In HET mice, the desmin mRNA levels were found to be higher than in WT animals, but lower than in HOM mice. An analogous pattern was detected for syncoilin, whereas in case of synemin mRNA, only HOM mice showed an up-regulation. Mean values and standard errors were obtained by fourfold repeated measurements in nine (desmin, syncoilin) or four (synemin) 3-month-old animals per genotype. *Columns* represent relative values with wild-type expression scaled to 1. *p* values were calculated using the Kruskal–Wallis one-way analysis of variance; post hoc analyses were performed using the Mann–Whitney *U* test. **d** Synemin and syncoilin immunoblotting of lysates from soleus muscle. While the synemin protein levels were slightly increased in HET and unchanged HOM mice, the expression of syncoilin was significantly reduced in both R349P desmin genotypes. GAPDH was used as loading control. **e**, **f** Densitometry analyses of synemin and syncoilin immunoblots. Column charts were generated from analyses of 16 sets of synemin and 9 sets of syncoilin WT, HET, and HOM bands and show relative values with wild-type expression scaled to 1. *p* values were calculated using the Kruskal–Wallis one-way analysis of variance; post hoc analyses were performed using the Mann–Whitney *U* test

The observed alterations in the subcellular distribution of desmin and its direct binding partners are associated with changes in the respective mRNA and protein expression levels. QRT-PCR analyses demonstrated a marked up-regulation of desmin mRNA in HOM R349P knock-in mice (Fig. 6a). Taken into account that the desmin protein level is dramatically reduced in this genotype, these results indicate a highly increased protein turnover of the mutant desmin. Moreover, HOM R349P desmin knock-in mice showed markedly increased synemin and syncoilin mRNA levels (Fig. 6b, c). In HET animals, the total desmin and syncoilin mRNA levels were found to be higher than in WT, but lower than in HOM animals. On the protein level, the expression of syncoilin was markedly reduced in both R349P desmin genotypes, whereas the synemin levels were seemingly unchanged (Fig. 6d–f).

### From pathology of the extrasarcomeric cytoskeleton to muscle weakness and increased mechanical vulnerability of skeletal muscle fibers

**Fig. 7:**
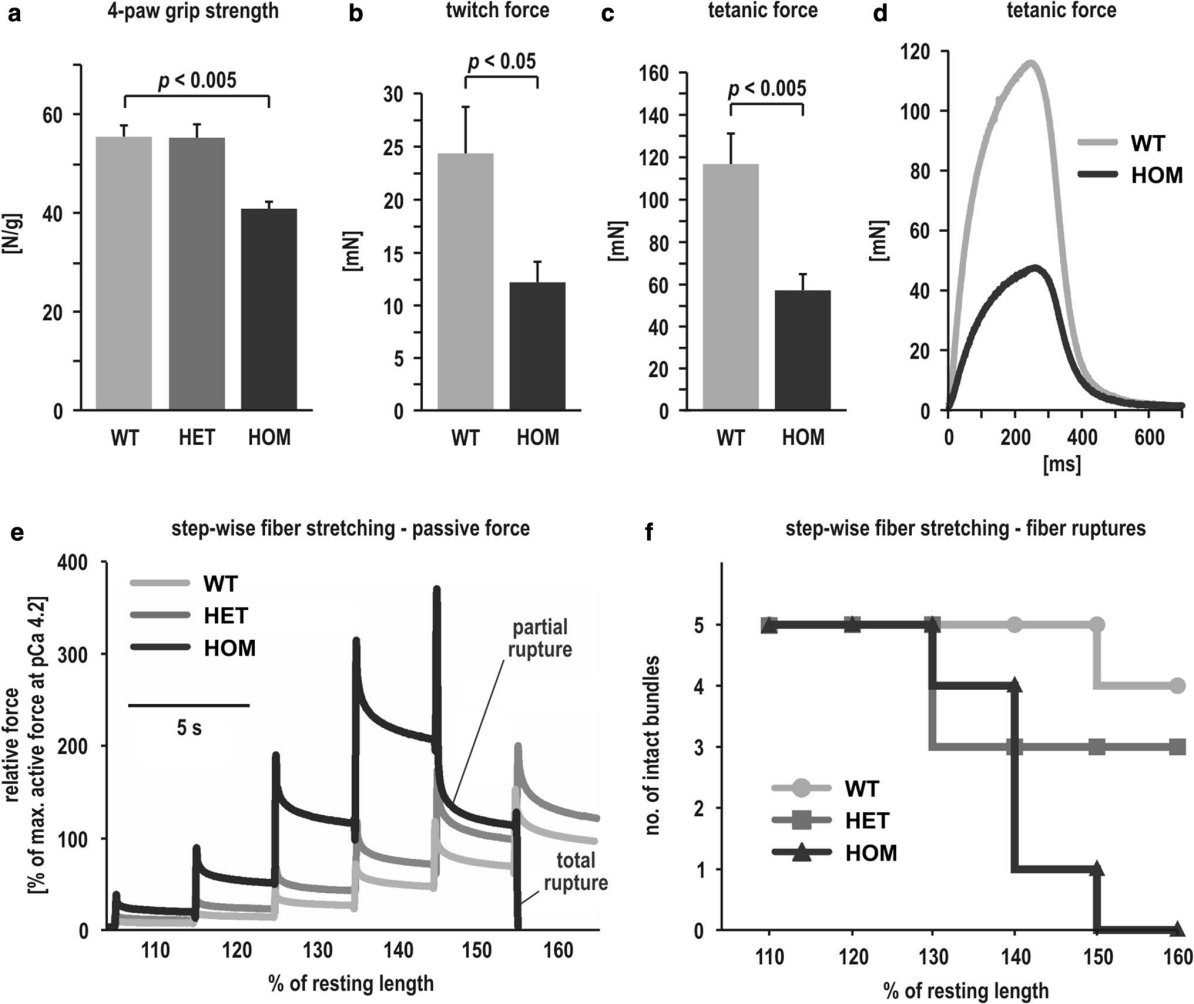
From skeletal muscle weakness to increased mechanical vulnerability of isolated muscle fibers. **a** Analyses of 19-month-old R349P desmin knock-in mice revealed a significant reduction of the 4-paw grip strength in HOM mice (*n* = 8), whereas HET mice (*n* = 2) displayed values matching those of WT animals (*n* = 7). *Columns *represent mean values from five measurements per animal, *error bars* indicate standard errors of the mean, **b**–**d** soleus muscles from some of these animals (WT, *n* = 6, HET, *n* = 2, HOM, *n* = 7) were explanted and subjected to twitch (**b**) and tetanic force (**c**) recordings. *Columns *represent averaged peak values from ten measurements per muscle, *error bars* indicate standard errors of the mean. **d** Typical myograph curves of tetanic force development during electrostimulation. **a**–**d** since we were only able to include two HET animals they were excluded from statistical evaluations; statistical significance of WT vs. HOM was calculated using the Lord test. **e** Representative example recordings of passive force during step-wise increase in stretch of 5-fiber bundles from soleus muscles of 4.5-month-old mice. During each step-increase in stretch, restoration force quickly jumps to a maximum, followed by exponential force decay due to elastic relaxation of elastic elements in the bundles. Larger force responses indicate higher biomechanical stiffness, i.e., reduced elasticity. Note the increased stiffness in HET and, more markedly, in HOM animals. **f** The expression of R349P desmin leads to an increased mechanical vulnerability of the isolated muscle fiber bundles. While all five wild-type fibers and three out of five heterozygote fibers remained intact up to a resting length of 150 %, four out of five fibers derived from HOM animals were ruptured

To address clinically relevant skeletal muscle weakness, we performed a multi-level investigation. In a first step, animals were monitored in vivo by 4-paw grip strength measurements and wire hanging tests. Young animals up to an age of 12 months showed no significant differences between the genotypes (data not shown). The analysis of 1.5-year-old WT, HET, and HOM animals revealed a statistically significant reduction in the 4-paw grip strength in HOM mice as compared to WT mice (Fig. 7a). These animals also showed a highly pathological performance in the wire hanging test (WT, *n* = 5, 124.4 s vs. HOM, *n* = 8, 58.5 s; *p* < 0.005, Lord test). To further substantiate the observed changes in single muscle groups, soleus muscles from some of the above animals were explanted and subjected to twitch and tetanic force recordings. Homozygous muscles developed significantly reduced forces in both tests as compared to WT (Fig. 7b–d). Corresponding analyses were performed using explanted extensor digitorum longus muscles. Here, no significant changes in the force development were detected (data not shown).

To address the passive biomechanical properties of skeletal muscle fibers, chemically skinned small fiber bundles from soleus muscles derived from other, 4.5-month-old mice were step-wise stretched using an automated force transducer system. Five fiber bundles from HET and markedly more pronounced from HOM mice exhibited higher biomechanical stiffness, i.e., increased restoration force at identical extensions, than those from WT mice, indicating that the expression of mutant desmin reduced fiber elasticity (Fig. 7e). In addition, the fiber bundles from HOM mice, and to a lesser extent from HET mice, were prone to passive-stretch-induced fiber ruptures (Fig. 7f).

### From mutant desmin to dilated cardiomyopathy, cardiac conduction defects, and arrhythmias

Cardiac pathology comprising cardiomyopathy, conduction defects and arrhythmias significantly contributes to the increased mortality rate and reduced life expectancy in patients with autosomal dominant and recessive desminopathies [12]. Similarly, cardiac assessment in both HET and HOM R349P desmin knock-in mice revealed a combined picture of cardiomyopathy, conduction defects and arrhythmias. Functional cardiac magnetic resonance imaging (CMR) showed the typical signs of dilated cardiomyopathy with significantly reduced stroke volumes and ejection fractions in combination with increased end-systolic and end-diastolic volumes in 2-year-old HOM mice (Fig. S7). In vivo assessment of cardiac hemodynamics by pressure volume loop recordings with determination of d*P*/d*t*_max_ values revealed decreased myocardial contractility in 1-year-old HOM animals (Fig. S8a), whereas impaired contractility in HET animals could not be detected before the age of 2 years (Fig. S8b). Thus, in keeping with our morphological analysis, also aged HET knock-in animals developed a significant cardiomyopathy. The diagnosis of cardiomyopathy in these aged HET mice was further substantiated by a 1.9-fold increase in BNP mRNA expression levels (WT, *n* = 5, HET, *n* = 8, *p* < 0.05, Lord test).

Extensive electrophysiological investigations in young, 6-month-old R349P desmin knock-in mice demonstrated a broad spectrum of cardiac abnormalities already at this age. Long-term telemetric baseline electrocardiography recordings showed sinus rhythm in all animals. Atrial and ventricular extrasystoles could be registered in both mutant genotypes, whereas spontaneous atrial fibrillation (AF) or ventricular tachycardias (VT) were not detected (Fig. S9a, b). During stress tests, i.e., swimming exercise, the number of premature ventricular contractions significantly increased in mutant mice (Fig. S9c; WT, *n* = 3, 4.0 ± 6.9 events/h, HET, *n* = 3, 65.3 ± 51.4 events/h, HOM, *n* = 3, 21.0 ± 12.7 events/h; *p* < 0.0001, Chi-squared test). In addition, HET and HOM mice showed higher grade atrio-ventricular and sino-atrial blocks (Fig. S9d, e) under conditions of stress during the telemetry ECG recordings (WT, 0.5 ± 0.7 events/h, HET, 3.3 ± 0.6 events/h, HOM, 15.0 ± 4.2 events/h; *p* < 0.0012, Chi-squared test).

The surface electrocardiography recordings demonstrated no differences in cardiac conduction times, i.e., P-, PQ-, QRS-, and QTc-intervals. In a next step, 3-month-old animals underwent catheterization for intracardiac electrography for assessment of supra-Hisian AV nodal conductance (AH interval) and infra-Hisian conduction times (HV interval). This analysis demonstrated normal AH intervals, but significantly prolonged HV intervals in HET and HOM mice (Fig. S8c, d). Furthermore, we noted the occurrence of 2nd and 3rd degree AV blocks in HET and HOM mice, which never occurred in WT animals (Fig. S10a). Transvenous atrial and ventricular stimulation maneuvers further revealed increased numbers of episodes with AF and VTs in both genotypes (Fig. S8e, f). In contrast to WT animals, in which only short, self-terminating episodes of AF could be induced, both HET and HOM mice displayed long-lasting (>1 min) AF-episodes (Fig. S10b). Ventricular stimulations also led to a significant increase in the number of VT episodes in mutant mice (Fig. S10c).

## Discussion

The majority of IF diseases are caused by heterozygous missense mutations leading to pathogenic, single amino acid exchanges. Since almost all available antibodies do not allow a distinction between IF protein species that differ only by a single amino acid, the expression level, subcellular distribution, and turnover of wild-type and mutant IF protein species cannot be directly assessed in diseased human tissues or animal models. The creation of a R349P desmin knock-in mouse model expressing untagged mutant desmin in conjunction with the generation of a R349P mutant-specific antibody allowed us to analyze these central issues in relation to the clinical, hemodynamic, morphological, and biomechanical aspects of desminopathies.

### From in vitro to in vivo: how much mutant desmin is needed to cause protein aggregation pathology in striated muscle?

Our previous IF assembly assays demonstrated that presence of already 25 % of the human R350P desmin mutant in mixtures with wild-type desmin effectively ablated the normal polymerization process of desmin IFs [3]. These in vitro findings imply that the ratio of mutant versus wild-type desmin is a critical issue. We therefore studied the expression of both desmin mRNA and protein in human and murine striated muscle tissues. Our analysis of human R350P skeletal muscle revealed that 55 % of total desmin mRNA was derived from the mutant allele, whereas the values were 43 and 55 %, respectively, in skeletal and cardiac muscle tissue derived from HET R349P desmin knock-in mice. Though the relative amounts of mutant and wild-type desmin mRNA were seemingly balanced, our quantitative RT-PCR analysis revealed that the total levels of desmin mRNA were constantly increased in striated muscle tissue of our HET R349P desmin knock-in mice. In contrast, immunoblot analysis showed only 20 % of mutant desmin in total protein extracts derived from human R350P skeletal muscle, and a range of 20–50 % in striated muscle from HET R349P mice. Thus, our HET mice closely mirror the expression levels of mutant and wild-type desmin in heterozygous human R350P mutation carriers.

With regard to the pathophysiology of autosomal dominant desminopathies, our expression data in man and mice may provide a novel understanding of the results in two previously reported transgenic mouse models for desminopathies. In one model, the low expression rate of only 10 % of a HA-tagged L345P mutant desmin may explain the very mild clinical skeletal and cardiac muscle phenotypes as well as the lack of desmin aggregation pathology [24]. In contrast, a second model with the p.Arg173_Glu179del desmin transgene showed a threefold overexpression of the mutant protein. Though this model developed a progressive protein aggregate cardiomyopathy [47], such high expression levels of a mutant desmin do not occur in heterozygous human mutation carriers [12]. However, beyond the critical expression level of the mutant desmin, specific properties of the individual desmin mutations and their subcellular distributions are likely to contribute to the observed phenotypes.

In comparison to the desmin protein levels in WT and HET animals, our HOM R349P knock-in mice showed markedly reduced amounts of desmin. The virtual lack of desmin expression in the only two reported human autosomal recessive desminopathies, however, seems to be related to a different disease mechanism. In one case with a 22-base pair deletion in exon 6 causing a premature stop codon, dramatically reduced levels of both mutant desmin mRNA and protein were reported. Muscle biopsies from these patients showed a myopathic pattern, but no protein aggregates on the ultrastructural level [8]. In contrast to our HOM mouse model, the mutant desmin mRNA in the homozygous mutations carriers was dramatically reduced to <0.5 % possibly due to nonsense-mediated mRNA decay. In another patient with compound heterozygote truncation mutations (p.Thr76fsX21, p.Glu108X) a complete ablation of desmin protein was described [19]. Though no mRNA levels were reported in this study, the ablation of desmin protein also may be attributed to a nonsense-mediated mRNA decay.

The complete lack of wild-type desmin and the low amounts of mutant R349P desmin in our HOM R349P desmin knock-in mice further highlight and validate essential aspects of our previously reported transfection and in vitro assembly studies using human R350P desmin [3]. In line with these studies, our analysis of HOM R349P desmin knock-in animals clearly demonstrated that the desmin mutant itself is incapable of forming a desmin network and leads to the formation of desmin-positive protein aggregates in striated muscle cells. Thus, the lack of wild-type desmin and the intrinsic inability of the mutant desmin to form a functional desmin IF network are the primary key events in the molecular sequence that lead to progressive skeletal and cardiac muscle damage in HOM R349P desmin knock-in mice. However, as several desmin mutants have been reported which are capable of forming IF structures in vitro or in transfected cells [4], these mutants are likely to exert their pathogenic potential via other molecular mechanisms.

### Mutant versus wild-type desmin: increased protein turnover and segregation into aggregates

The desmin mRNA and protein expression data derived from our mice are indicative of an increased desmin turnover rate. To address this issue in more detail, we treated our mice with cycloheximide, which completely blocks protein synthesis. Immunoblot analysis of the desmin protein levels in WT, HET, and HOM mice demonstrated that the R349P mutant desmin disappeared much faster than the wild-type protein. This finding provides an explanation for the markedly reduced desmin protein levels in our HOM mice. Moreover, the presence of mutant desmin also induced an accelerated decay of wild-type desmin in HET mice.

The generation of a mutant-specific desmin antibody further enabled us for the first time to visualize the subcellular distribution of mutant desmin in human and murine striated muscle tissue. In man and mice, the mutant desmin was predominantly detected in protein aggregates in the subsarcolemmal region, and, to a lesser extent, in the sarcoplasm of skeletal muscle fibers. In cardiomyocytes of HET and HOM mice, the R349P desmin was primarily found at the level of intercalated discs. Importantly, the analysis of skeletal and cardiac muscle tissue from HET animals revealed that mutant desmin was not incorporated into the cross-striated, wild-type desmin cytoskeleton, but was segregated into subsarcolemmal and sarcoplasmic protein aggregates.

### From mutant desmin via pathology of the extrasarcomeric cytoskeleton to altered biomechanical properties of muscle fibers

We demonstrated that the expression of mutant desmin not only leads to an increased turnover of desmin protein species, but also increases the turnover or changes the subcellular distribution of synemin, syncoilin, and plectin, all of which are direct desmin-binding partners and key components of the extrasarcomeric cytoskeleton. Thus, mutant desmin ultimately damages the structural and functional organization of the three-dimensional extrasarcomeric IF cytoskeleton on multiple levels, however, with distinct differences between individuals carrying one or two mutated alleles. In heterozygous human and murine muscle tissue the IF pathology is characterized by focal disruptions of the IF network in a subset of fibers. Given the multi-nuclear nature of skeletal muscle fibers, these focal disruptions of the IF cytoskeleton in heterozygous man and mice may preferentially be triggered in areas with higher levels of mutant desmin protein.

In HOM R349P knock-in mice and autosomal recessive human desminopathies the pathology is clearly different. Here, no wild-type desmin is present and the disruption of the IF cytoskeleton is no longer focal but omnipresent in every striated muscle cell. The latter observation is clearly reminiscent of the findings in desmin knock-out mice, in which the lack of a desmin network leads to a progressive myopathy and cardiomyopathy [18, 25, 31]. However, in desmin knock-out mice the complete ablation of desmin neither altered the subcellular localization of plectin [7] nor led to protein aggregation pathology [25, 26, 30]. Moreover, desmin knock-out mice displayed calcifications in the cardiac tissue that were not present in our R349P desmin knock-in model [25, 30]. The comparison of the desmin knock-out and our HOM R349P knock-in mice highlights the importance of the structural and functional integrity of the extrasarcomeric IF cytoskeleton, but also implies that the mutant desmin exerts different biological effects than the pure lack of wild-type desmin.

In striated muscle, the desmin filament system has been attributed to play a cell-protective role against mechanical stress by dissipating mechanical energy during muscle contraction [12]. To address the question if the observed extrasarcomeric IF pathology alters the passive biomechanical properties of skeletal muscle fibers in our R349P desmin knock-in mice, we performed analyses on chemically skinned small fiber bundles from soleus muscles, which were step-wise stretched. Here, we were able to demonstrate a higher restoration force reflecting a higher stiffness and an increased susceptibility to passive-stretch-induced ruptures of muscle fibers from both genotypes. These findings basically mirror the results of our biomechanical analysis of primary myoblasts derived from a patient with a heterozygous R350P desmin mutation, which displayed increased cell stiffness and a higher rate of cell death and substrate detachment [6]. Our data demonstrates for the first time that the presence of mutant desmin protein changes the functional properties of the extrasarcomeric cytoskeleton and subsequently alters the biomechanical properties of striated muscle fibers. The observed increase of stiffness is likely to inflict changes in the adaptability of striated muscle cells to mechanosensing and mechanotransduction, and may thus essentially contribute to the progressive skeletal and cardiac muscle damage in desminopathies.

### Desmin protein aggregate pathology: a matter of age and muscle groups

Our mutant-specific desmin antibody proved to be invaluable for the detection of an age- and muscle group-dependent protein aggregation pathology in HET R349P desmin knock-in mice. We observed a dramatic increase in number and size of desmin-positive protein aggregates in aged mice in comparison to young animals. Moreover, we demonstrated that the protein aggregation pathology in soleus muscle was much more abundant than in gastrocnemius or quadriceps femoris muscles. The former muscle is mainly composed of slow-twitch type 1 fibers with a high content of mitochondria, whereas the latter two are mainly composed of fast-twitch type 2 fibers. Taken into account that marked dystrophic changes and severe concomitant mitochondrial abnormalities were only present in soleus muscles of HOM mice, these findings strongly imply that expression of mutant desmin is more harmful for slow-twitch muscle fibers.

### Pathogenic significance of desmin protein aggregates in striated muscles

Desmin-positive protein aggregates are the morphological hallmark of desminopathies and all other MFMs. However, it is an open debate if, and to what extent, these protein aggregates contribute to the development of progressive muscle damage and weakness. In the present study, we demonstrate that marked desmin-positive protein aggregation can be present without concomitant myopathological alterations or skeletal muscle weakness. Thus, our data strongly imply that total or focal disruption of the extrasarcomeric IF cytoskeleton rather than the presence of desmin protein aggregates per se is the key factor that triggers the progressive muscle fiber damage in autosomal dominant and recessive desminopathies.

### Desminopathies in man and mice: similarities and differences

In contrast to both previously published transgenic desminopathy mouse models [24, 47], our knock-in approach has the striking advantage that the expression of the mutant desmin gene remains under control of the endogenous gene regulation sites. Thus, our HET animals express the R349P mutant desmin in the physiological range that we detected in heterozygous human mutation carriers. As in humans, our HOM animals with the omnipresent extrasarcomeric cytoskeletal pathology display an earlier and more severe disease manifestation as HET mice, in which the disruption of three-dimensional IF network was more focal in nature. Desmin-positive protein aggregates, the morphological hallmark of desminopathies and all other forms of MFMs, were present in both HET and HOM mice, but clear evidence of progressive skeletal muscle weakness and morphological signs of myopathic alterations was only present in the latter. In human heterozygous mutation carriers, the R350P desmin mutation is associated with marked clinical variability ranging from severely to only mildly affected patients with different clinical presentations of skeletal muscle weakness [3, 46]. Though we cannot rule out that the observed level of mutant R349P desmin protein in our HET knock-in mice is principally not sufficient to induce progressive skeletal muscle damage, we presume that the limited physical activity of our sedentary laboratory mice basically prevents the development of muscle weakness and damage.

In contrast, the cardiological workup demonstrated all three desmin-related cardiac disease manifestations in HET and HOM mice, i.e., true cardiomyopathy, conduction defects, and arrhythmias. As in humans, our mice displayed high incidence of AV conduction blocks, which was accompanied by a marked prolongation of the infra-Hisian AV time indicating reduced electrical impulse propagation in the Purkinje fiber network. Since desmin is the most abundant protein in these fibers [45], the expression of mutant desmin may significantly disturb the cellular function of the cardiac conduction system. This might explain the high susceptibility of desminopathy patients to life-threatening conduction defects. As in R350P desmin mutation carriers sudden cardiac death is frequently occurring, partially independent from the onset of severe heart failure symptoms [46], such potentially asymptomatic patients have to be surveyed comprehensively and early implantation of a pacemaker or implantable cardioverter–defibrillator (ICD) may be indicated. Furthermore, the high incidence of VTs appearing in the R349P knock-in mice may be attributed to the presence of dilated cardiomyopathy and the interstitial myocardial fibrosis [17].

### From R349P desmin knock-in mice towards therapeutic implications

To date, no specific or ameliorating therapy is available for desminopathies and all other forms of MFMs. Even the basic clinical question if physical activity would be beneficial or harmful for affected patients cannot be answered on the basis of the available clinical and scientific literature. Our observation that the structural and functional disturbances of the extrasarcomeric cytoskeleton led to an increased susceptibility of muscle fibers to mechanical strain implicates that in patients with desminopathies physical activity beyond a certain threshold or duration might aggravate the skeletal or cardiac disease progression. Our insights into the molecular pathogenesis also put a focus on pharmacological compounds that inhibit the increased IF protein turnover we observed in our R349P desmin knock-in mice. If effective, they might ameliorate the decay of the extrasarcomeric cytoskeleton in some autosomal dominant desminopathies, facilitate segregation of mutant desmin into harmless protein aggregates, and thus slow down the disease progression. Here, it is tempting to speculate that proteasome inhibitors, which already found their way into clinical practice, could be promising therapeutic drugs for some desmin mutation carriers protecting the extrasarcomeric cytoskeleton from degradation.

## Electronic supplementary material

Below is the link to the electronic supplementary material.

Supplementary material 1 (DOCX 57 kb)

Supplementary material 2 (TIFF 812 kb)

Supplementary material 3 (TIFF 108 kb)

Supplementary material 4 (TIFF 3824 kb)

Supplementary material 5 (TIFF 6591 kb)

Supplementary material 6 (TIFF 1880 kb)

Supplementary material 7 (TIFF 2398 kb)

Supplementary material 8 (TIFF 1303 kb)

Supplementary material 9 (TIFF 136 kb)

Supplementary material 10 (TIFF 368 kb)

Supplementary material 11 (TIFF 87 kb)
