# Supplementary material for: The toxic effect of R350P mutant desmin in striated muscle of man and mouse
Source: Acta Neuropathol. 2014 Nov 14;129(2):297–315. doi: 10.1007/s00401-014-1363-2 (PMC4309020; doi:10.1007/s00401-014-1363-2)
Supplement: Supplementary file 1 — Supplementary material 1 (DOCX 57 kb) [file 401_2014_1363_MOESM1_ESM.docx]

**Supplemental Figure Legends**

**Supplemental Figure 1**

**Structural organization of the desmin molecule and desmin gene targeting strategy a** Illustration of a human desmin monomer with black boxes representing α-helical coiled coil segments 1A, 1B, and 2, the connecting linker segments L1 and L12, and the N-terminal head and C-terminal tail domains. Positions of the amino acid stretch encoded by exon 6 as well as the R350P missense mutation are indicated. Note, that the murine desmin lacks one amino acid, serine 82, as compared to the human protein. **b** Scheme of the targeting strategy resulting in the R349P desmin knock-in mice at the genomic level. **c** PCR genotyping employing the indicated primer pair confirms the presence of a knock-in allele with a 244 bp product containing the loxP site vs. 179 bp derived from the wild-type allele. **d** Southern blot verification of knock-in mice based on NsiI restriction digestion and hybridization with an external 5’ probe leading to the detection of the expected 4.4 kb knock-in and 6.0 kb wild-type fragments. **e** Verification of the presence of the R349P desmin mutation at the genomic level by sequencing of the indicated 1,755 and 1,820 bp PCR products from HET mice; the chromatogram shows the expected double signal for AGG (Arg) and CCC (Pro) from the wild-type and knock-in alleles, respectively.

**Supplemental Figure 2**

**Aberrant migration pattern of human R350P mutant desmin in SDS-PAGE.** **a** Recombinant human wild-type and R350P mutant desmin were expressed in BL21 *E. coli* and Sf9 insect cells, and analyzed by immunoblotting. Note, that the mutant desmin displayed the same slower migration pattern as observed in human and murine tissues. **b** Protein extracts from cardiac muscle tissue derived from WT, HET, and HOM desmin knock-in mice were subjected to *in vitro* dephosphorylation assays using alkaline phosphatase (AP). Note, that no decrease in the higher apparent molecular mass of mutant desmin could be observed.

**Supplemental Figure 3**

**Cardiac fibrosis in R349P desmin knock-in mice.** **a** Hematoxylin and eosin (H&E) and Sirius red stains reveal areas of increased connective tissue in 5-month-old HET and, more prominent, in HOM mice. **b** The column chart displays the extent of fibrosis as percent area of extracellular Sirius red-staining in left (LV) and right (RV) ventricles of WT, HET, and HOM mice. Number of animals, WT n = 19, HET n = 22, HOM n = 16; columns, mean values; error bars, standard errors of the mean; statistical analysis, 1-way ANOVA with Bonferroni subgroup analysis.

**Supplemental Figure 4**

**Subcellular distribution of wild-type and mutant desmin in human and murine skeletal muscle tissue.** **a** Analysis of human normal and **b**, R350P desminopathy skeletal muscle tissue. **a** Typical desmin immunostaining in a cross-section of normal human skeletal muscle using the commercially available mouse monoclonal desmin antibody (pan-desmin). **b** Upper row, typical desminopathy staining pattern with predominantly subsarcolemmal and also sarcoplasmic desmin-positive protein aggregates using the pan-desmin antibody as well as our R350P desmin mutation-specific antibody. **b** Lower row, higher magnification of a single muscle fiber containing multiple protein aggregates. Note the massive alterations of the extrasarcomeric desmin cytoskeleton in which wild-type and mutant desmin display a partial separation in their subcellular distribution. The latter finding indicates that the mutant desmin is not incorporated into the normal desmin network. **c,d** Corresponding desmin immunofluorescence images from soleus muscle derived from 3- **c** and 16-month-old **d** R349P desmin knock-in mice. **c** Upper row, using the pan-desmin antibody no obvious differences between WT and HET mice could be detected. In contrast, the HOM animals displayed a highly pathological pattern with several fibers containing multiple desmin-positive aggregates and other fibers, in which the sarcoplasmic desmin signal is markedly reduced. **c** Lower row, and **d**, the desmin mutant-specific antibody revealed an age-related accumulation of pathological desmin-positive aggregates in HET and HOM mice. **e** Pan-desmin and R349P desmin stains in longitudinal sections of murine gastrocnemius muscle. Note the regular cross-striated desmin pattern (pan-desmin) in WT mice, which is mainly preserved in HET mice. In contrast, the R350P desmin is only present in pathological protein aggregates. In keeping with the observation in human skeletal muscle, the latter finding underlines a separation of the mutant desmin from the wild-type desmin filament system.

**Supplemental Figure 5**

**Subcellular localization of wild-type and mutant desmin in cardiac muscle tissue and isolated cardiomyocytes from R349P desmin knock-in mice.** **a** Detection of wild-type and mutant desmin in cardiac muscle tissue from WT, HET and HOM animals using the pan-desmin and the R349P desmin mutant-specific antibodies. In contrast to WT and HET mice, which display the characteristic desmin immunolabelling at Z-discs and intercalated discs, HOM animals showed highly abnormal desmin localization with increased immunoreactivity at intercalated discs and a virtually complete loss of the cross-striated pattern. In addition, small dotted desmin-positive protein aggregates were visible inside the sarcoplasm of individual cardiomyocytes. **b** Detection of wild-type and mutant desmin in isolated cardiomyocytes from WT, HET and HOM animals using the pan-desmin and the R349P desmin mutant-specific antibodies. Corresponding to the analysis of cardiac muscle tissue, desmin immunolabelling was highly abnormal in homozygous cardiomyocytes. Here, the R349P mutant desmin is highly enriched at the level of intercalated discs in combination with absent cross-striated pattern. Higher magnification of intercalated disc regions showed that in contrast to wild-type and heterozygous cardiomyocytes, which displayed a filamentous organization of desmin, homozygous R349P desmin cardiomyocytes showed abnormal intercalated disc regions with non-filamentous, dotted desmin-positive protein aggregates. Specific detection of the R349P mutant desmin in heterozygous cardiomyocytes demonstrates that the mutant protein was virtually absent within the sarcoplasm, but enriched at the intercalated disc regions.

**Supplemental Figure 6**

**Altered subcellular distribution of the direct desmin-binding partner synemin in skeletal muscle tissue of a human desminopathy.** **a** In normal human gastrocnemius muscle, the direct desmin interaction partner synemin shows a sarcoplasmic distribution. **b** In a R350P desminopathy synemin instead accumulated in the subsarcolemmal region of muscle fibers.

**Supplemental Figure 7**

**Functional cardiac MRI depicts a dilated cardiomyopathy in aged homozygous R349P desmin knock-in mice. a** Representative cardiac MRI images from 2-year-old WT and HOM animals during end-diastole and end-systole. Sax, short axis view; 4cv, 4-chamber view. **b** Statistical analyses of the ejection fractions, end-diastolic, end-systolic, and stroke volumes of the left ventricles for WT (n = 6), HET (n = 6) and HOM (n = 5) R349P desmin knock-in mice were done using the Kruskal–Wallis one-way analysis of variance; post hoc analyses were performed using the Mann–Whitney U test; columns, mean values; error bars, standard errors of the mean. The HOM mice showed a clear impairment of left ventricular function with significant reductions of the ejection fraction and stroke volume as well as a significant increase of the end-systolic volume (the increase of end-diastolic volume just failed to reach statistical significance).

**Supplemental Figure 8**

**Dilated cardiomyopathy, conduction defects, and arrhythmias in R349P desmin knock-in mice.** **a** Recordings of pressure-volume loops by catheterization of 1-year-old WT (n = 5), HET (n = 8), and HOM (n = 8) mice provided clear evidence of dilated cardiomyopathy in HOM animals (reduced myocardial contractility as assessed by reduced maximal rate of pressure development in the left ventricle, dP/dt_max_ value). Statistical significance was calculated using the Kruskal–Wallis one-way analysis of variance; post hoc analyses were performed using the Mann–Whitney U test; error bars indicate standard errors of the mean. **b** Note, that in 2-year-old HET mice (n = 8 vs. n = 5 WT animals) assessment of pressure-volume loops also provided evidence for a significant impairment of left ventricular contractility. Thus, aged HET mice also develop a dilated cardiomyopathy. Statistical significance was calculated using the Lord test; error bars indicate standard errors of the mean. **c,d** Assessment of supra-Hisia (compact AV node) conductance (AH interval) and infra-Hisian (specific conduction system) conductance (HV interval) by intracardiac electrography. Though the surface electrocardiography showed normal PQ-times and the intracardiac electrography normal AH intervals, the HV intervals were significantly prolonged in HET and HOM mice. **e,f** Transvenous atrial and ventricular stimulation maneuvers revealed a trend towards increased numbers of atrial fibrillation (AF) episodes and ventricular tachycardias (VTs) in HET and HOM mice. **c-f** Number of animals, WT, n = 19, HET, n = 22, HOM, n = 16; statistical significance was calculated by 1-way ANOVA with Bonferroni subgroup analysis; error bars indicate standard errors of the mean.

**Supplemental Figure 9**

**Cardiac conduction abnormalities and arrhythmias in R349P desmin knock-in mice visualized by telemetric long-term electrocardiography.** **a** Representative electrocardiography recording from a WT littermate exhibiting sinus rhythm without any sign of ectopy or arrhythmia throughout the whole registration period. **b-d** Examples of electrocardiography recordings in 6-month-old HET mice displaying spontaneous ectopic P-waves (arrows), polymorphic premature ventricular contractions (PVCs, arrowheads), and AV blocks (asterisks indicate the continuously running P-waves). The PVCs seen in **c** were recorded during a physical stress test (10 min swimming exercise). **e** Representative electrocardiography recording from a 6-month-old HOM littermate. In addition to polymorphic PVCs these animals exhibited extended AV blocks up to 414 ms. Note, that the unsteady isoelectric line results from excessive movement during the stress test. The cycle lengths (ms) between subsequent QRS complexes are indicated. Number of investigated animals, WT, n = 3, HET, n = 3, HOM, n = 3.

**Supplemental Figure 10**

**R349P desmin knock-in mice show conduction defects and increased susceptibility to induction of prolonged arrhythmias.** **a** Catheterization for intracardiac electrography and transvenous atrial and ventricular stimulation maneuvers of 3-month-old mice resulted in the occurrence of 2^nd^ and 3^rd^ degree AV blocks in HET and HOM mice, which were never detected in WT mice. **b** In contrast to WT animals, in which only short, self-terminating episodes of atrial fibrillation could be induced, both HET and HOM mice displayed long-lasting (> 1 min) episodes. **c** Ventricular stimulations led to a significant increase in the number of episodes with ventricular tachycardia in HET and HOM mice in the subgroup of inducible animals. Number of animals, WT, n = 19, HET, n = 22, HOM, n = 16; mean values and standard errors of the mean are shown; statistical analyses, **a** Fisher's exact test, **b** Chi-squared test, **c** 1-way ANOVA with Bonferroni subgroup analysis.

**Supplemental Materials and Methods**

**Generation and genotyping of R349P desmin knock-in mice**

The R349P desmin knock-in mouse model B6J.129Sv-*Des*^tm1(R349P)Cscl&Rfsr^ was generated according to our specifications (CSC, RS) by genOway, Lyon, France. For construction of the targeting vector a floxed neomycin resistance cassette was flanked from 5’ to 3’ by a 1.5 kbp region of the desmin gene comprising exons 3 to 6, the latter including the R349P point mutation, and a 3.9 kbp region comprising exons 7 to 9, respectively. Linearized targeting vector was transfected into 129Sv ES cells (1x10^8^ ES cells, 100 µg linearized plasmid, 260 V, 500 µF). Positive selection was started 48 h after electroporation by addition of 200 µg/ml of G418 (150 µg/ml of active component, Life Technologies GmbH, Darmstadt, Germany). 1,281 resistant clones were isolated, amplified in 96-well plates, and duplicates were made. The set of plates containing ES cell clones amplified on gelatin were genotyped by both PCR (spanning the 5’ homology region) and Southern blot (internal and external probes on both 3’ and 5’ ends) analyses; PCR products were sequenced in order to validate the presence of the R349P point mutation. One clone (13-2E) was identified as correctly targeted at the *Des* locus, microinjected into C57BL/6J blastocysts, and gave rise to male chimeras with a significant ES cell contribution (agouti coat color), which were bred to C57BL/6J mice expressing Cre recombinase to remove the neomycin cassette.

Routine genotyping of the resulting R349P desmin knock-in mouse strain was performed by PCR (primer pair 5'-AAACCTGGAAGCAGTTTTACACAAGAGGC-3' and 5'-GCTGTAGGTTTTTAATTCTAAAGGTGGATAAGGG-3'), resulting in products of 179 bp for the wild-type and 244 bp for the R350P desmin knock-in allele. In addition, mice were genotyped at random by Southern blotting using a 5’ external probe, which gives rise to a 6.0 kb signal for the wild-type and a 4.4 kb signal for the knock-in allele. Further, the presence of the R350P desmin point mutation at the genomic level was verified at random by PCR (primer pair 5’-TCTCTGGTCCCCACTTGAGCTGTTC-3’ and 5’-CATGGCTCCCTTGACTGGCAGTAAT-3’) in conjunction with sequencing of the 1,755 and 1,820 bp products derived from the wild-type and knock-in alleles, respectively.

Mice were housed in isolated ventilated cages (IVC) equipped with spruce granulate embedding and a nest under specific and opportunistic pathogen-free (SOPF) conditions at a temperature of 22 ± 2 °C, an air humidity of 50 to 70 %, a ventilation rate of 70 air exchanges per hour, and a light-dark-cycle of 12/12 hrs with free access to water and food. Littermates were separated at weaning by sex and housed at a maximum of five animals per cage. Health monitoring was done as recommended by the Federation of European Laboratory Animal Science Associations (FELASA).

Mice were handled in accordance with the German Animal Welfare Act (Tierschutzgesetz) as well as the German Regulation for the protection of animals used for experimental purposes or other scientific purposes (Tierschutz-Versuchstierverordnung), and the investigations were approved by the responsible governmental animal care and use office (North Rhine-Westphalia State Agency for Nature, Environment and Consumer Protection (LANUV), Recklinghausen, Germany; reference number 8.87-50.10.31.09.045).

**Investigation of muscle strength**

Grip strength was measured using a BIO-GS3+ grip strength meter (Bioseb, Vitrolles, France). Animals were allowed to grasp the rectangular ring with their 4 paws and were pulled horizontally until the grip was released. For the wire hanging test mice were placed on a wire cage lid, which was lightly shaken causing the mouse to grip the wire. The lid was slowly turned upside down (180°) and the latency to fall was recorded for the maximum of 300 s. For determination of twitch and tetanic force recordings soleus muscles of WT and desmin knock-in mice were explanted, immersed in Ringer solution (118 mM NaCl, 3.4 mM KCl, 0.8 mM MgSO_4_, 1.2 mM KH_2_PO_4_, 11.1 mM glucose, 25 mM NaHCO_3_, 2.5 mM CaCl_2_) and bubbled with carbogen (95 % O_2_, 5 % CO_2_) at room temperature. Muscles were stretched to an optimal length and electrostimulation was performed with a field electrode as described previously [30]. Ten single consecutive twitches were recorded with a train of stimuli (20 V, 1 ms) at 0.1 Hz. Tetanic forces were recorded by ten consecutive tetani at a frequency of 75 Hz with a train of stimuli at 0.006 Hz (approx. 1 tetanus per 3 minutes).

**Preparation of isolated cardiomyocytes**

Murine cardiomyocytes were isolated according to [27]. Excised hearts were prepared in Tyrode’s solution with EGTA instead of CaCl_2_ (135 mM NaCl, 4 mM KCl, 1 mM MgCl_2_, 2 mM HEPES, 2.6 mM EGTA, 10 mM glucose, 1 mg/ml BSA, pH 7.4) and mounted in a Langendorff perfusion system. Pressure was adjusted to 0.05 bar and the temperature to 36 °C. Hearts were perfused with the preparation solution for 5 min followed by a high-K^+^ solution for 5 min (4 mM NaCl, 10 mM KCl, 130  mM K-glutamate, 1 mM MgCl_2_, 0.05 mM CaCl_2_, 2 mM HEPES, 10 mM glucose, 1 mg/ml BSA, pH 7.4). Trypsin (1,000 BAE units/40 ml; Roche, Mannheim, Germany) and collagenase (type L, 25 mg in 40 ml, Sigma-Aldrich, St. Louis, MO, USA) were added to the high-K^+^ solution, and hearts were perfused for 8 to 10 min in trypsin and for another 10 to 13 min in collagenase. Hearts were then cut into small parts, transferred into Tyrode’s solution with 1.8 mM CaCl_2_ in place of EGTA and supplemented with 0.17 mg/ml trypsin-inhibitor (Sigma-Aldrich), the pieces were disintegrated by stirring with glass rods, and finally the suspension was filtered and gently centrifuged.

**Analysis of biomechanical properties of skeletal muscle fibers**

Small fiber bundles of five single fibers were dissected from soleus muscles of HET and HOM R349P desmin knock-in mice as well as WT littermates. Muscles were immersed in Ca^2+^-buffered solutions (Ca^2+^-free) and transferred to an automated force transducer system for skinned fiber bundle recordings. Bundles were attached to the pin of a force transducer (KG-7, Scientific Instruments, Heidelberg, Germany) and a counter-pin fixed to a voice coil actuator. To obtain diffusional access to the sarcoplasm, fiber bundles were shortly immersed in saponin-containing relaxing solution [9]. For passive stretch experiments, the voice coil actuator was driven via a custom-written LabView program to quickly stretch the bundle from the resting length L_0_ (bundle adjusted to slack length) to step-wise length increases in 10 % bins, which were held for 5 s before continuing with stretching. The force was recorded via a Wheatstone-based bridge amplifier calibrated for output voltage. Typically, restoration force sharply increase immediately with stretch, which is followed by an exponential decline in force to a plateau due to relaxation of elastic elements [13]. The stretch length at which rupture of the bundle occurred was noted and analyzed from five bundles each to construct survival curves with stretch.

**Functional cardiac MRI**

Functional cardiac MRI (CMR) of the ventricle was performed in HET and HOM R349P desmin knock-in mice as well as WT littermates. All data were acquired with a 4-element cardiac phased-array coil on an 11.7 T small animal system (BioSpec 117/16, Bruker, Ettlingen, Germany), applying a self-gated imaging technique (IntraGate, Bruker, Ettlingen, Germany [12]). During reconstruction, 20 cardiac phases were generated at spatial resolution of 117² x 500 µm³. Ejection fractions (EF), end-diastolic (EDV), end-systolic (ESV), and stroke volumes (SV) were calculated from the functional MRI data using Segment (MEDVISO, Lund, Sweden [29]).

**Transthoracic echocardiography**

The ventral area of mice was depilated one day before echocardiography. For echocardiography, a Sonos 5500 (Philips, Eindhoven, The Netherlands) with a S12 transducer (12 MHz) was used, and the performing person was blinded during procedure as previously described [21]. To evaluate cardiac function, three consecutive beats were used to obtain the left ventricular end-diastolic diameter (LVEDD) and the left ventricular end-systolic diameter (LVESD). The cardiac function is represented as the decline in fractional shortening (FS) which was calculated as FS [%] = [(LVEDD-LVESD)/LVEDD] x 100. Furthermore, the thickness of left ventricular posterior wall in diastole (PWTd) was measured.

**Recording of pressure volume loops**

Measurements were made in closed-chest, spontaneously breathing mice as previously described [3, 21]. A 1.2-French catheter (Model FT111B, SciSense Inc., London, ON, Canada) was inserted into the left ventricle of the mouse through the carotid artery to simultaneously measure pressure and volumes. Left ventricular volumes were extrapolated from admittance magnitude and admittance phase in real-time using the ADVantage PV system (SciSense Inc.). Pressure and volume data were recorded using a Scisense FA-404 four-channel 16-bit A/D converter recorder with LabScribe2 software (SciSense Inc.) [21].

**Real-time polymerase chain reactions**

For analysis of mRNA expression levels of brain natriuretic peptide (BNP) total RNA was extracted from cardiac tissue lysates of HET and HOM R350P desmin knock-in and WT control mice with the RNeasy Fibrous Tissue Mini Kit (Qiagen, Hilden, Germany). SuperScript III reverse transcriptase (Invitrogen, Karlsruhe, Germany) was used to randomly reverse transcribe 1 µg of RNA into cDNA according to the manufacturer’s specifications. Quantitative real-time PCR was performed on an ABI PRISM 7000 (Applied Biosystems, Foster City, USA) using the QuantiTect Primer Assays #QT00107541 (Qiagen, Hilden, Germany) and SYBR GreenER qPCR SuperMix (Invitrogen, Karlsruhe, Germany) [21]. Analyses of the expression levels of desmin and desmin-binding partners was done using striated muscle cDNA prepared as described below and the QuantiTect SYBR Green PCR Kit (Qiagen) on a CFX Connect Real-Time PCR Detection System (Bio-Rad Laboratories, Hercules, CA, USA) or an Opticon II instrument (MJ Research Inc., St. Bruno, Quebec, Canada) in 96-well plates according to the manufacturer’s protocol. Primer pairs preferably spanning exon-exon junctions were designed using the NCBI tool at http://www.ncbi.nlm.nih.gov/tools/primer-blast/; product sizes were 100 to 300 bp, melting temperatures 58 to 62 °C. Possible self-complementarity and hairpin formation of the primers were analyzed using the OligoCalc tool at http://www.basic.northwestern.edu/biotools/oligocalc.html. For quality control of the PCR products, a melting curve analysis was performed after the 40^th^ or 50^th^ cycle from 65 to 95 °C in 0.5 °C steps for 0.5 s. Moreover, C_t_ values of a negative control were determined. Further, selected reaction mixtures were analyzed by agarose gel electrophoresis. For data analysis, the delta-delta-C_q_-Method (ΔΔC_q_) was used, which is based on normalization with a single reference gene. The difference in C_q_ values (ΔC_q_) between the target gene and the reference gene is calculated, the C_q_ values of the different samples are directly compared [5], and the expression ratios are displayed as fold change in relation to the wild-type control samples. Primer pairs used were 5’-ACCACAGTCCATGCCATCAC-3’ and 5’-TCCACCACCCTGTTGCTGTA-3’ for GAPDH (housekeeping), 5’-AAGCTTGCTGGTGAAAAGGA-3’ and 5’-TTGCGCTCATCTTAGGCTTT-3’ for HPRT (housekeeping), 5’-GCTCCGTTATAGATGACAGC-3’ and 5’-ATCTCGATGTGGAAGATGAG-3’ for AIP1, 5’-GAGGCTGAAGAATGGTACAA-3’ and 5’-CTCATCCTTTAGGTGTCGGA-3’ for desmin, and QuantiTect Primer Assays #QT01044904 and #QT00129178 (Qiagen) for synemin and syncoilin, respectively.

**Surface electrocardiography**

A surface 6-lead electrocardiogram was continuously monitored and analyzed under stable conditions for three minutes [2]. Incidence of ventricular and supraventricular ectopic beats was qualitatively evaluated for the whole observation time. Data were amplified, filtered, sampled at 2 kHz, and digitally stored (LabSystem, C. R. Bard Inc., New Jersey, USA). The rate corrected QT-interval (QTc) was calculated according to [18].

**Long-term electrocardiography recording**

For long-term electrocardiography analysis in conscious animals, telemetry devices (Modell EA-F20; DataSciences International, St. Paul, MN, USA) were implanted with the use of a sterile technique [10]. Surgery was performed under inhalation anesthesia (induction period 2.5 vol. %, maintenance 1.2 vol. % isoflurane in 70 % N_2_O/30 % O_2_) and intraperitoneal anesthesia with ketamine hydrochloride (0.033 mg/g). After performing a midline incision on the back along the spine the implantable 3.5 g wireless radiofrequency transmitter was inserted into a subcutaneous tissue pocket. The leads were directed ventrally and fixed to the pectoral muscle in an Eindhoven II position, and skin incisions were sutured. Recordings were performed 10 days after recovery from surgical instrumentation. All baseline recordings were performed in conscious animals for 24 h in a constant environment. Electrocardiography signals were recorded with the use of a telemetry receiver (PhysioTel Receiver RPC-1, DataSciences International, St. Paul, MN, USA) and a 12-bit A/D converter (Powerlab 8/30, AD Instruments, Dunedin, New Zealand) at a sampling rate of 1 kHz. Standard criteria were used to measure electrocardiogram parameters [18]. At the end of the baseline recording a physical stress test, i.e., 10 min swimming exercise, was performed under continuation of electrocardiogram recording as described before [14].

**Intracardiac electrophysiological investigation**

*In vivo* transvenous electrophysiological investigations were performed using a single catheter technique [20]. Preparation, catheterization, and electrophysiological investigation were performed under inhalation anesthesia (induction period 2.5 vol. %, maintenance 1.2 vol. % isoflurane in 70 % N_2_O/30 % O_2_). After preparation of the jugular vein a 2-French octapolar mouse electrophysiological catheter (eight 0.5 mm circular electrodes, electrode-pair spacing 0.5 mm; Ciber Mouse, NuMed Inc., NY, USA) was positioned in the right cardiac cavities on atrial and ventricular level. Intracardiac electrograms and transvenous atrial and ventricular stimulation maneuvers were registered and recorded as previously described [20]. Intracardiac electrocardiograms were analyzed for AH (interval from first atrial signal to His signal) and HV (interval from His to first intracardiac ventricular signal) times as surrogates for supra- and infra-Hisian conductivity, respectively. Performing fixed-rate pacing using a modified multi-programmable stimulator (Model 5328; Medtronic, MN, USA), sinus node recovery time (SNRT), Wenckebach periodicity (WBP), and 2:1 AV nodal block were evaluated. SNRT was defined as maximum return cycle length after 10 s fixed-rate pacing at S1S1 cycle length of 120 ms. WBP and 2:1 AV nodal block were calculated by fixed rate atrial pacing (10 s fixed rate at S1S1 of 120 ms, 5 ms step-wise reduction). WBP was defined as longest S1S1 cycle length with loss of 1:1 AV nodal conduction, 2:1-block as longest S1S1 with 2:1 AV nodal conduction. Atrial and AV nodal refractory periods (ARP and AVNRP) were evaluated by programmed atrial stimulation (7 stimuli fixed rate at S1S1 cycle length of 120 ms, one short coupled extrastimulus with a 5 ms step-wise S1S2 reduction). AVNRP was defined as longest S1S2 with loss of AV nodal conduction, ARP as longest S1S2 with absent atrial response. Ventricular refractory period (VRP) was evaluated similarly to ARP by ventricular extrastimulus pacing. The inducibility of atrial fibrillation (AF) was tested by atrial burst stimulation (5 s at S1S1 of 50-10 ms, 10 ms step-wise reduction, stimulus amplitudes 1 and 2 mA). AF was defined as rapid and fragmented atrial electrograms with irregular AV nodal conduction for ≥1 s [22]. Ventricular vulnerability was tested by ventricular burst stimulation (1 s at S1S1 of 50-10 ms, 10 ms step-wise reduction, stimulus amplitudes 1 and 2 mA). Ventricular tachycardia (VT) was defined as ≥4 ventricular ectopic beats.

**Human skeletal muscle biopsy material**

Tissue samples of skeletal muscle derived from a diagnostic muscle biopsy of a patient from a previously reported family with a heterozygous R350P desmin mutation [31] were obtained from the Friedrich-Baur-Institute, Munich, Germany.

**Preparation of striated muscle cryosections, immunohistochemistry, and immunofluorescence stains**

Skeletal and cardiac muscle specimens were collected and immediately frozen in liquid nitrogen-cooled isopentane. Cryostat sections of 5 µm thickness were collected on microscope slides and air-dried for 30 min. Histology was performed using a routine staining protocol and an Olympus CX41 light microscope (Olympus, Hamburg, Germany). For immunohistochemistry, transversal and longitudinal sections were fixed for 5 min with acetone, air-dried for 30 min, and permeabilized with PBS containing 0.2 % Triton X-100 for 15 min. Non-specific binding was blocked with 10 % fetal calf serum, 1 % goat serum and 0.1 % sodium azide in PBS for 1 h at room temperature. Incubation with primary antibodies diluted in PBS with 3 % BSA was done overnight at 4 °C or for 1 h at room temperature. After washing, sections were incubated with Alexa-fluor anti-mouse and anti-rabbit goat antibodies (1:200, Molecular Probes/Life Technologies GmbH, Darmstadt, Germany), and finally washed with PBS and mounted in Mowiol for analysis using a Leica TCS SP5/AOBS/tandem scanning system (Leica Microsystems GmbH, Wetzlar, Germany) with emission detection in sequential mode equipped with the Leica LAS-AF software (v. 2.6.0.7266).

**Ultrastructural analysis**

For transmission electron microscopy, soleus muscle specimens were fixed in 2 % glutaraldehyde in 0.1 M phosphate buffer, pH 7.2, postfixed in 2 % buffered osmium tetroxide, dehydrated in graded alcohol concentrations, and embedded in epoxy resin according to standard protocols. 1 µm semi-thin sections for orientation were stained with toluidine blue. Ultra-thin sections were stained with uranyl acetate and lead citrate, and examined with a LEO906E transmission electron microscope (Carl Zeiss GmbH, Oberkochen, Germany).

**Quantitation of myocardial fibrosis**

Four micrometer sections of paraffin embedded myocardial whole heart samples were dewaxed with ethanol and stained with Sirius red (0,1 % in saturated aqueous picric acid) [4]. Myocardial fibrosis was determined in 10 random fields of the left and right ventricles in a four-chamber-view section of the heart at 200x magnification. Cardiac fibrosis was defined as the percent area of extracellular Sirius red-staining and computed with the use of a digital image analysis program (Adobe Photoshop, Adobe Systems Corporation, San Jose, CA, USA).

**Preparation of RNA and reverse transcriptase reaction**

For extraction of total RNA from striated muscle 20 to 50 mg of snap-frozen tissue were pulverized in a mortar on liquid nitrogen before addition of 1 ml Trizol and thawing to room temperature. After 5 min incubation, 200 µl CHCl_3_ were added, and the samples were incubated for further 3 min and centrifuged for 10 min at 12,000 g and 4 °C. Supernatants were transferred into new reaction tubes, 500 µl isopropanol were added, and the mixtures were incubated for 10 min at room temperature followed by another centrifugation step. The RNA pellets were washed once with 75 % ethanol, air dried, dissolved in 20 µl of RNAse free water by 10 min incubation at 55 °C, aliquoted and stored at -80 °C. Quality of RNA was determined using a 2100 Bioanalyzer (Agilent Technologies, Santa Clara, CA, USA), and quantity using a NanoDrop 1000 Spectrophotometer (Thermo Fisher Scientific Inc., Waltham, MA, USA) or a NanoQuant plate in conjunction with an Infinite M1000 plate reader (Tecan, Männedorf, Switzerland). For synthesis of cDNA 1 µg of each RNA was transcribed randomly-primed employing the M-MLV (M1705; Promega Corporation, Madison, WI, USA) or Superscript III (Qiagen, Hilden, Germany) reverse transcriptase.

**Determination of wild-type and R350P/R349P mutant desmin mRNA levels**

Complementary DNA derived from striated muscle of a R350P desminopathy patient or HET R349P desmin knock-in mice was used for PCR (primer pair, human: 5’-CTGAAGCTGAGGAGTGGTAC-3’ and 5’-TTGAGCAGGTCCTGGTACTC-3’, product 310 bp; primer pair, mouse: 5’-GAGGCTGAAGAATGGTACAA-3’ and 5’-CTCATCCTTTAGGTGTCGGA-3’, product 270 bp). To analyze the level of wild-type and mutant desmin mRNA, PCR products either were directly subjected to restriction digestion or first cloned into pGEM-Teasy (Promega Corporation, Madison, WI, USA) for transformation into *E. coli*, growth of colonies, use for colony PCR, and then subjected to restriction digestion. As the desmin point mutation destroys an endogenous AciI restriction site in human (digestion of wild-type derived PCR product into 179 and 131 bp fragments) and it leads to insertion of a novel AvaI restriction site in mouse (digestion of mutant derived PCR product into 92 and 178 bp fragments), the amounts of wild-type and mutant desmin mRNA can be determined by densitometry (digestion of direct, mixed PCR product) or counting the clones (digestion of colony PCR product).

**Antibodies**

Wild-type R350/R349 desmin specifically was detected by rabbit pAb HD2 (1:1,000 in TBS-T for western blotting), and R350P/R349P mutant desmin by rabbit pAb HD350P (1:1,000 in TBS-T for western blotting, 1:200 in PBS for immunofluorescence). Both antibodies were newly generated for the purpose of this study by immunization of rabbits with hepta-peptides surrounding amino acid residue 350/349 of desmin (wild-type peptide C-MRQMREL, mutant peptide C-MRQMPEL; PSL Peptide Specialty Laboratories GmbH, Heidelberg, Germany) and affinity purified.

Both wild-type and R350P/R349P mutant desmin were detected by three commercially available “pan-desmin” antibodies (D1033, Sigma-Aldrich (St. Louis, MO, USA), mouse mAb, 1:400 in TBS-T with 5 % milk powder for western blotting; D33, Dako (Glostrup, Denmark), mouse mAb, 1:400 in PBS-T for western blotting, 1:50 or 1:100 in PBS for immunofluorescence; 10570, Progen Biotechnik GmbH (Heidelberg, Germany), rabbit pAb, 1:100 in PBS for immunofluorescence), GAPDH by a mouse mAb (G9295, Sigma-Aldrich (St. Louis, MO, USA), 1:50,000 in PBS for western blotting), synemin isoform 2 by a rabbit pAb ([33], 1:500 in PBS-T for western blotting, 1:100 in PBS for immunofluorescence), syncoilin by rabbit pAbs (#2991 [17], 1:500 in PBS-T for western blotting; Bs-9463R, Bioss Inc. (Woburn, MA, USA), 1:1,000 in PBS-T for western blotting) and a goat pAb (S-14, sc-162284, Santa Cruz Biotechnology Inc. (Dallas, TX, USA), 1:50 in PBS for immunofluorescence), plectin by a rabbit pAb (#9, [1], 1:3,000 in PBS-T for western blotting) and a guinea pig pAb (GP21, Progen Biotechnik GmbH (Heidelberg, Germany), [23], 1:400 in PBS for immunofluorescence).

**SDS-PAGE of samples from striated muscle tissue**

For reproducible immunoblotting extraction of proteins from striated muscle tissue was done according to [6]. Small amounts of snap-frozen tissue were pulverized in a mortar on liquid nitrogen before addition of lysis buffer (5 mM Tris, 10 % SDS, 0.2 M DTT, 1 mM EDTA, pH 6.8), boiling at 95 °C for 5 min, sonicating for 10 s, again boiling at 95 °C, and centrifugating of the lysate at 13,000 g for 10 min. Supernatants were used undiluted for protein quantitation using a fluorometric dye (ProStain, Active Motif, Carlsbad, CA, USA), and 1:4 diluted with 1x SDS sample buffer (25 mM Tris, 0.8 % SDS, 2 % 2-mercaptoethanol, 4 % glycerol, 0.001 % bromophenol blue, pH 6.8) and again boiled for gel electrophoresis and western blotting.

**Two-dimensional gel electrophoresis, in vitro dephosphorylation, and mass spectrometry**

2D-SDS-PAGE was performed according to [7] using 18 cm Immobiline DryStrips (GE Healthcare, Munich, Germany) with linear or non-linear pH gradients from 4-7 or 3-10; second dimension SDS-PAGE was done using 12 % acrylamide gels followed by either Imperial Blue-staining (Pierce/Thermo Fisher Scientific Inc., Waltham, MA, USA) and picking of spots for mass spectrometry or immunoblotting. *In vitro* dephosphorylation assays were done as described in [32]. For mass spectrometry proteins were identified after in-gel digestion with V8 or trypsin protease by LC-MS/MS employing a HCT ETD II iontrap mass spectrometer equipped with a nano ESI source (Bruker Daltonics, Bremen, Germany) as described in [8].

**Expression of recombinant desmin in E. coli and insect cells**

For expression of recombinant human wild-type and R350P mutant desmin, BamHI/BglII desmin cDNAs were amplified by PCR from available plasmids using primer pair 5’-CGGGATCCGCCACCATGCATCACCATCACCATCACCATCACAGCCAGGCCTACTCGTCC-3’ and 5’-GAAGATCTTTAGAGCACTTCATGCTGCTG-3’, cloned into pTriEx1.1-Neo, which contains a bacterial T7, a mammalian CMV, and an insect cell baculovirus p10 promoter for expression of the gene of interest, and verified by sequencing. For expression of desmin protein in *E. coli* BL21(DE3)pLysS cells [26] and in insect cells Sf9 cells were used. For transfection of the latter, pTriEx1.1-Neo with desmin insert was mixed with baculovirus DNA and GeneJuice transfection reagent according to the manufacturer’s protocol (Novagen/Merck, Darmstadt, Germany); virus amplification was done for four days, before the viral supernatant was collected for infection of a fresh Sf9 culture and expression of desmin.

**Immunofluorescence analyses of isolated cardiomyocytes**

Isolated ventricular cardiomyocytes in Tyrode’s solution (see above) were allowed to attach to laminin-coated microscope slides for 90 min. In some preparations Tyrode’s solution was supplemented with wheat germ agglutinin to decorate the cell membrane [25] (1:20 WGA conjugated to Alexa Fluor 647; Life Technologies GmbH, Darmstadt, Germany). Cells were fixed with acetone/methanol 1:1 at -20 °C for 10 min. After evaporation of acetone/methanol the specimens were rehydrated in PBS. Non-specific binding sites were blocked by 10 % new born goat serum (NGS) + 1 % BSA in PBS at 37 °C for 20 min. Primary antibody incubation was performed over night at 4 °C, followed by three washes in PBS. Anti-rabbit IgG donkey conjugated to Alexa Fluor 488 (1:500, Molecular Probes/Life Technologies GmbH, Darmstadt, Germany) was applied at 37 °C for 3 h. DNA was stained with DAPI 1:16,000 in PBS at RT for 5 min. Images were recorded using a Leica TCS SP5/AOBS/tandem scanning system (Leica Microsystems GmbH, Wetzlar, Germany) with emission detection in sequential mode equipped with the Leica LAS-AF software (v. 2.6.0.7266).

**Cycloheximide assay**

For effective blockade of protein synthesis mice received daily s. c. injections at their neck of 60 mg/kg cycloheximide, typically 180 µl of a 10 mg/ml solution in PBS, for 4 days (d1 to d4); first injections (d0) were with PBS only. Control mice daily only received PBS. Mice were killed by cervical dislocation, and tissues were dissected and snap-frozen in liquid nitrogen for further analyses. The administered dose of cycloheximide was derived from [11, 15, 16, 19, 24, 28] and ChemID*plus* at http://chem.sis.nlm.nih.gov/chemidplus/ (LD50 mouse s.c.: 160 mg/kg).

**Supplemental References**

1. Andrä K, Lassmann H, Bittner R, Shorny S, Fassler R, Propst F, Wiche G (1997) Targeted inactivation of plectin reveals essential function in maintaining the integrity of skin, muscle, and heart cytoarchitecture. Genes Dev 11:3143-3156

2. Baker LC, London B, Choi BR, Koren G, Salama G (2000) Enhanced dispersion of repolarization and refractoriness in transgenic mouse hearts promotes reentrant ventricular tachycardia. Circ Res 86:396-407

3. Bauer R, Macgowan GA, Blain A, Bushby K, Straub V (2008) Steroid treatment causes deterioration of myocardial function in the δ-sarcoglycan-deficient mouse model for dilated cardiomyopathy. Cardiovasc Res 79:652-661

4. Bozkurt B, Kribbs SB, Clubb FJ, Jr., Michael LH, Didenko VV, Hornsby PJ, Seta Y, Oral H, Spinale FG, Mann DL (1998) Pathophysiologically relevant concentrations of tumor necrosis factor-alpha promote progressive left ventricular dysfunction and remodeling in rats. Circulation 97:1382-1391

5. Bustin SA, Benes V, Garson JA, Hellemans J, Huggett J, Kubista M, Mueller R, Nolan T, Pfaffl MW, Shipley GL, Vandesompele J, Wittwer CT (2009) The MIQE guidelines: minimum information for publication of quantitative real-time PCR experiments. Clin Chem 55:611-622

6. Chopard A, Pons F, Charpiot P, Marini JF (2000) Quantitative analysis of relative protein contents by western blotting: comparison of three members of the dystrophin-glycoprotein complex in slow and fast rat skeletal muscle. Electrophoresis 21:517-522

7. Clemen CS, Fischer D, Roth U, Simon S, Vicart P, Kato K, Kaminska AM, Vorgerd M, Goldfarb LG, Eymard B, Romero NB, Goudeau B, Eggermann T, Zerres K, Noegel AA, Schröder R (2005) Hsp27-2D-gel electrophoresis is a diagnostic tool to differentiate primary desminopathies from myofibrillar myopathies. FEBS Lett 579:3777-3782

8. Clemen CS, Tangavelou K, Strucksberg KH, Just S, Gaertner L, Regus-Leidig H, Stumpf M, Reimann J, Coras R, Morgan RO, Fernandez MP, Hofmann A, Muller S, Schoser B, Hanisch FG, Rottbauer W, Blumcke I, von Horsten S, Eichinger L, Schröder R (2010) Strumpellin is a novel valosin-containing protein binding partner linking hereditary spastic paraplegia to protein aggregation diseases. Brain 133:2920-2941

9. Friedrich O, Yi B, Edwards JN, Reischl B, Wirth-Hucking A, Buttgereit A, Lang R, Weber C, Polyak F, Liu I, von Wegner F, Cully TR, Lee A, Most P, Volkers M (2014) Interleukin-1α Reversibly Inhibits Skeletal Muscle Ryanodine Receptor: A Novel Mechanism for Critical Illness Myopathy? Am J Respir Cell Mol Biol:in press

10. Gehrmann J, Hammer PE, Maguire CT, Wakimoto H, Triedman JK, Berul CI (2000) Phenotypic screening for heart rate variability in the mouse. Am J Physiol 279:H733-740

11. Gold PE, Sternberg DB (1978) Retrograde amnesia produced by several treatments: evidence for a common neurobiological mechanism. Science 201:367-369

12. Henderson M, De Waele L, Hudson J, Eagle M, Sewry C, Marsh J, Charlton R, He L, Blakely EL, Horrocks I, Stewart W, Taylor RW, Longman C, Bushby K, Barresi R (2013) Recessive desmin-null muscular dystrophy with central nuclei and mitochondrial abnormalities. Acta Neuropathol 125:917-919

13. Horowits R (1992) Passive force generation and titin isoforms in mammalian skeletal muscle. Biophys J 61:392-398

14. Knollmann BC, Kirchhof P, Sirenko SG, Degen H, Greene AE, Schober T, Mackow JC, Fabritz L, Potter JD, Morad M (2003) Familial hypertrophic cardiomyopathy-linked mutant troponin T causes stress-induced ventricular tachycardia and Ca^2+^-dependent action potential remodeling. Circ Res 92:428-436

15. Longo BM, Mello LE (1997) Blockade of pilocarpine- or kainate-induced mossy fiber sprouting by cycloheximide does not prevent subsequent epileptogenesis in rats. Neurosci Lett 226:163-166

16. Malcolm RE, Hiley CR (1984) Short term reductions in cerebral muscarinic receptor concentration of the mouse after in vivo administration of cycloheximide. Biochem Pharmacol 33:1605-1610

17. McCullagh KJ, Edwards B, Kemp MW, Giles LC, Burgess M, Davies KE (2008) Analysis of skeletal muscle function in the C57BL6/SV129 syncoilin knockout mouse. Mamm Genome 19:339-351

18. Mitchell GF, Jeron A, Koren G (1998) Measurement of heart rate and Q-T interval in the conscious mouse. Am J Physiol 274:H747-751

19. Morales-Corraliza J, Mazzella MJ, Berger JD, Diaz NS, Choi JH, Levy E, Matsuoka Y, Planel E, Mathews PM (2009) In vivo turnover of tau and APP metabolites in the brains of wild-type and Tg2576 mice: greater stability of sAPP in the beta-amyloid depositing mice. PLoS One 4:e7134

20. Roell W, Lewalter T, Sasse P, Tallini YN, Choi BR, Breitbach M, Doran R, Becher UM, Hwang SM, Bostani T, von Maltzahn J, Hofmann A, Reining S, Eiberger B, Gabris B, Pfeifer A, Welz A, Willecke K, Salama G, Schrickel JW, Kotlikoff MI, Fleischmann BK (2007) Engraftment of connexin 43-expressing cells prevents post-infarct arrhythmia. Nature 450:819-824

21. Schinkel S, Bauer R, Bekeredjian R, Stucka R, Rutschow D, Lochmuller H, Kleinschmidt JA, Katus HA, Muller OJ (2012) Long-term preservation of cardiac structure and function after adeno-associated virus serotype 9-mediated microdystrophin gene transfer in mdx mice. Hum Gene Ther 23:566-575

22. Schrickel JW, Bielik H, Yang A, Schimpf R, Shlevkov N, Burkhardt D, Meyer R, Grohe C, Fink K, Tiemann K, Luderitz B, Lewalter T (2002) Induction of atrial fibrillation in mice by rapid transesophageal atrial pacing. Basic Res Cardiol 97:452-460

23. Schröder R, Warlo I, Herrmann H, van der Ven PF, Klasen C, Blumcke I, Mundegar RR, Furst DO, Goebel HH, Magin TM (1999) Immunogold EM reveals a close association of plectin and the desmin cytoskeleton in human skeletal muscle. Eur J Cell Biol 78:288-295

24. Squire LR, Barondes SH (1972) Variable decay of memory and its recovery in cycloheximide-treated mice. Proc Natl Acad Sci U S A 69:1416-1420

25. Stegemann M, Meyer R, Haas HG, Robert-Nicoud M (1990) The cell surface of isolated cardiac myocytes--a light microscope study with use of fluorochrome-coupled lectins. J Mol Cell Cardiol 22:787-803

26. Studier FW, Moffatt BA (1986) Use of bacteriophage T7 RNA polymerase to direct selective high-level expression of cloned genes. J Mol Biol 189:113-130

27. Tiemann K, Weyer D, Djoufack PC, Ghanem A, Lewalter T, Dreiner U, Meyer R, Grohe C, Fink KB (2003) Increasing myocardial contraction and blood pressure in C57BL/6 mice during early postnatal development. Am J Physiol 284:H464-474

28. Trakatellis AC, Montjar M, Axelrod AE (1965) Effect of cycloheximide on polysomes and protein systhesis in the mouse liver. Biochemistry 4:2065-2071

29. Trombitas K, Wu Y, McNabb M, Greaser M, Kellermayer MS, Labeit S, Granzier H (2003) Molecular basis of passive stress relaxation in human soleus fibers: assessment of the role of immunoglobulin-like domain unfolding. Biophys J 85:3142-3153

30. Ursu D, Sebille S, Dietze B, Freise D, Flockerzi V, Melzer W (2001) Excitation-contraction coupling in skeletal muscle of a mouse lacking the dihydropyridine receptor subunit gamma1. The Journal of Physiology 533:367-377

31. Walter MC, Reilich P, Huebner A, Fischer D, Schröder R, Vorgerd M, Kress W, Born C, Schoser BG, Krause KH, Klutzny U, Bulst S, Frey JR, Lochmüller H (2007) Scapuloperoneal syndrome type Kaeser and a wide phenotypic spectrum of adult-onset, dominant myopathies are associated with the desmin mutation R350P. Brain 130:1485-1496

32. Xavier CP, Rastetter RH, Blomacher M, Stumpf M, Himmel M, Morgan RO, Fernandez MP, Wang C, Osman A, Miyata Y, Gjerset RA, Eichinger L, Hofmann A, Linder S, Noegel AA, Clemen CS (2012) Phosphorylation of CRN2 by CK2 regulates F-actin and Arp2/3 interaction and inhibits cell migration. Sci Rep 2:241

33. Xue ZG, Cheraud Y, Brocheriou V, Izmiryan A, Titeux M, Paulin D, Li Z (2004) The mouse synemin gene encodes three intermediate filament proteins generated by alternative exon usage and different open reading frames. Exp Cell Res 298:431-444
