# Supplementary figures and images for: The toxic effect of R350P mutant desmin in striated muscle of man and mouse
Source: Acta Neuropathol. 2014 Nov 14;129(2):297–315. doi: 10.1007/s00401-014-1363-2 (PMC4309020; doi:10.1007/s00401-014-1363-2)

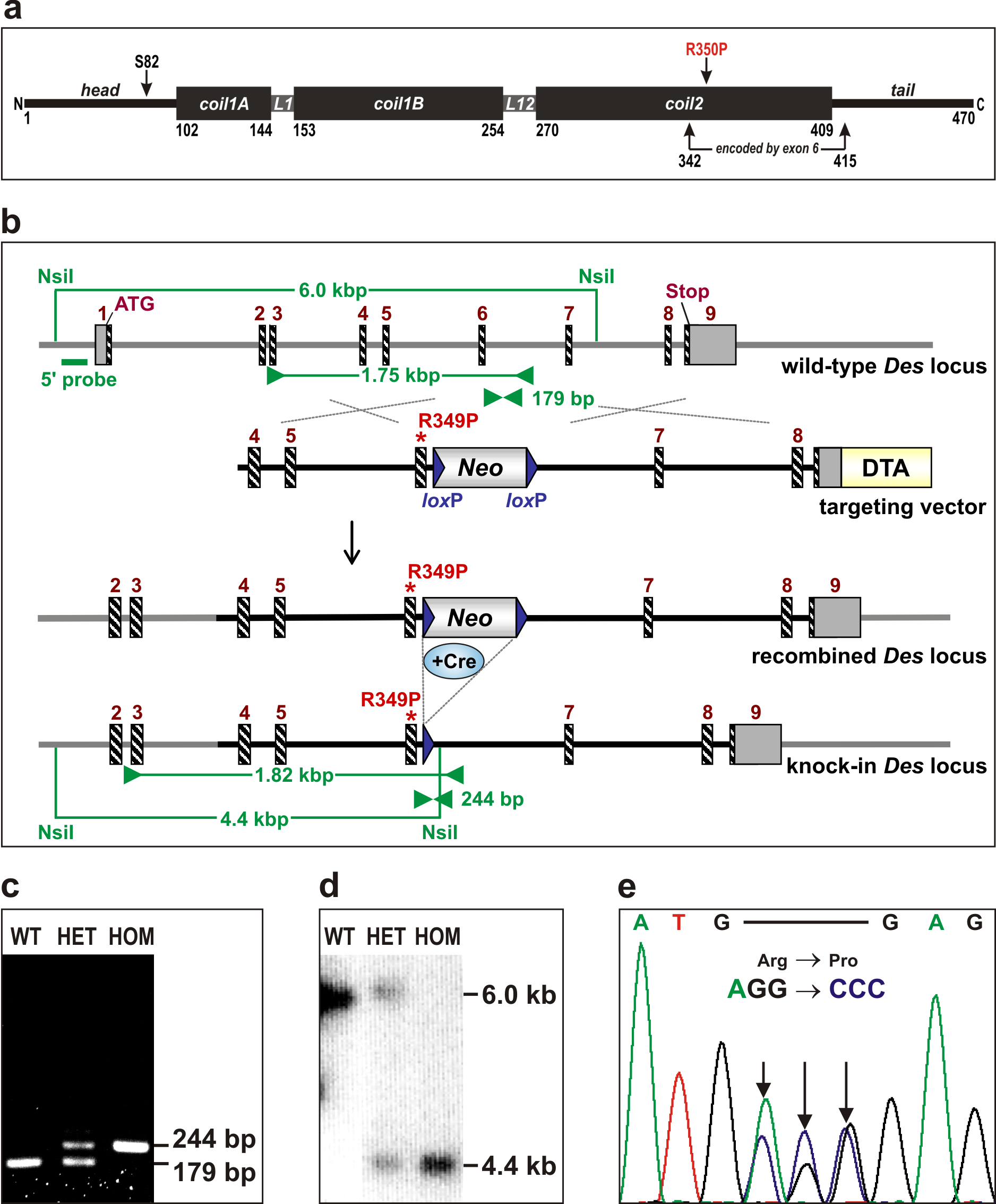

Supplement: Supplementary file 2 — Supplementary material 2 (TIFF 812 kb) [file 401_2014_1363_MOESM2_ESM.tif]

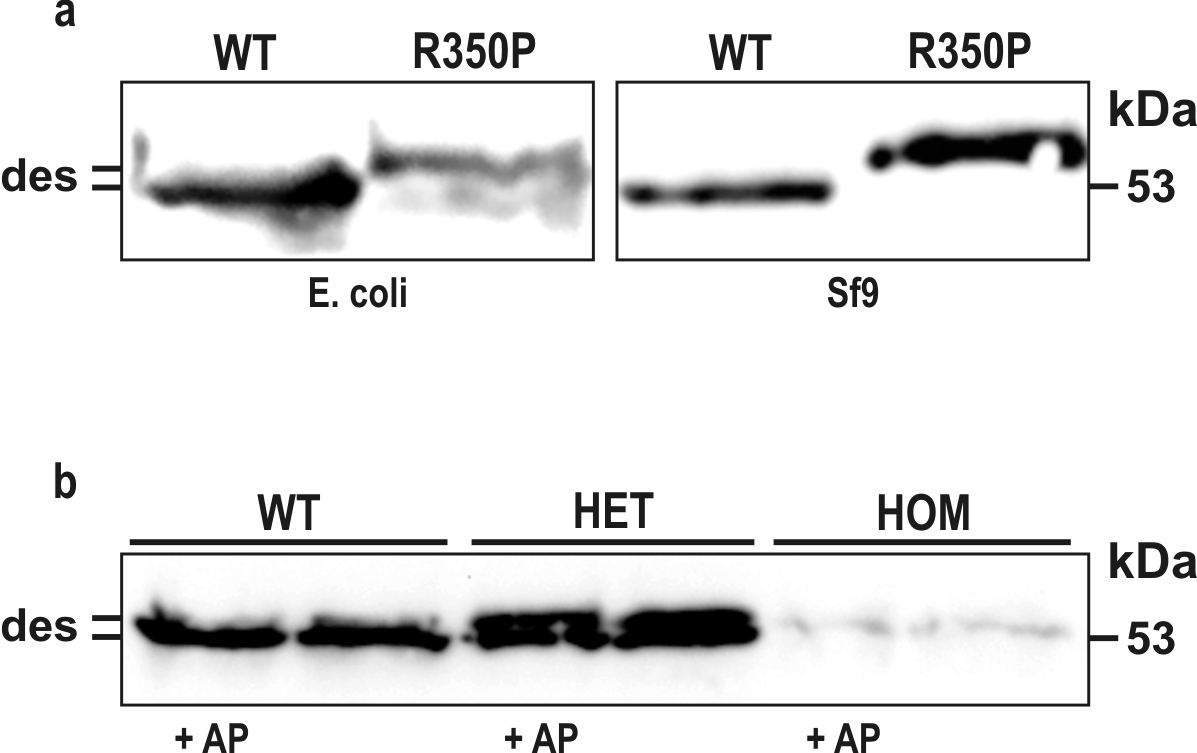

Supplement: Supplementary file 3 — Supplementary material 3 (TIFF 108 kb) [file 401_2014_1363_MOESM3_ESM.tif]

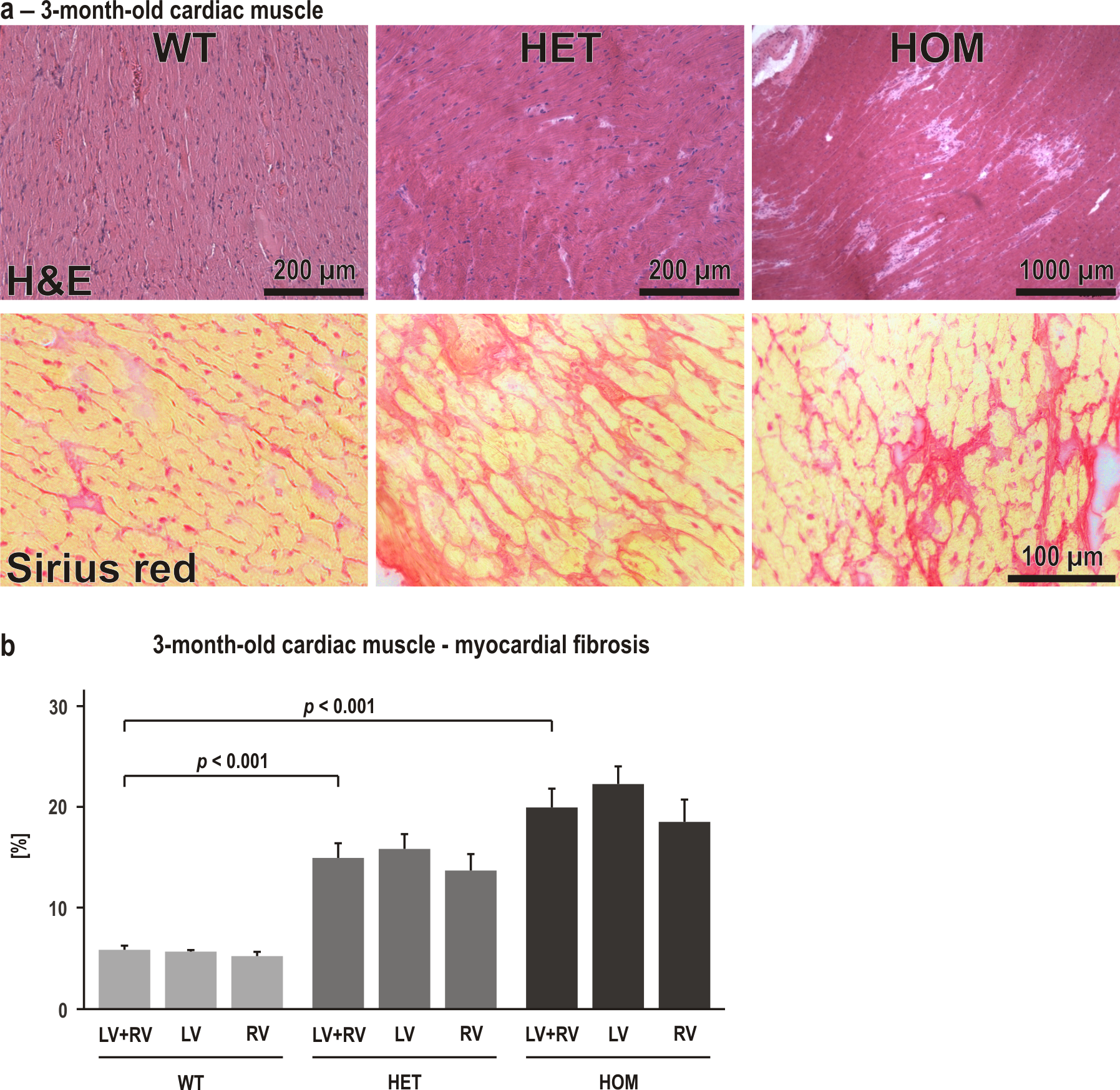

Supplement: Supplementary file 4 — Supplementary material 4 (TIFF 3824 kb) [file 401_2014_1363_MOESM4_ESM.tif]

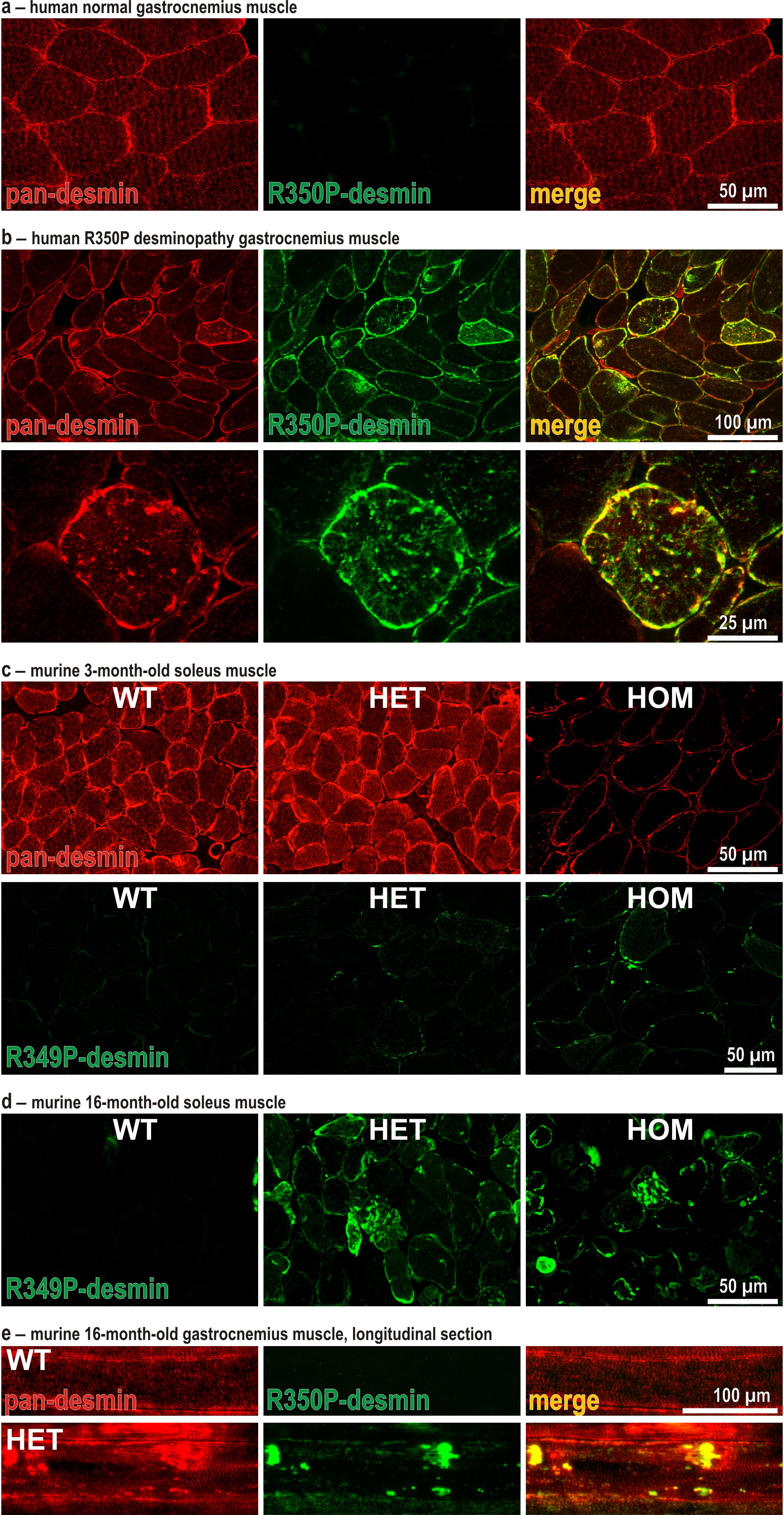

Supplement: Supplementary file 5 — Supplementary material 5 (TIFF 6591 kb) [file 401_2014_1363_MOESM5_ESM.tif]

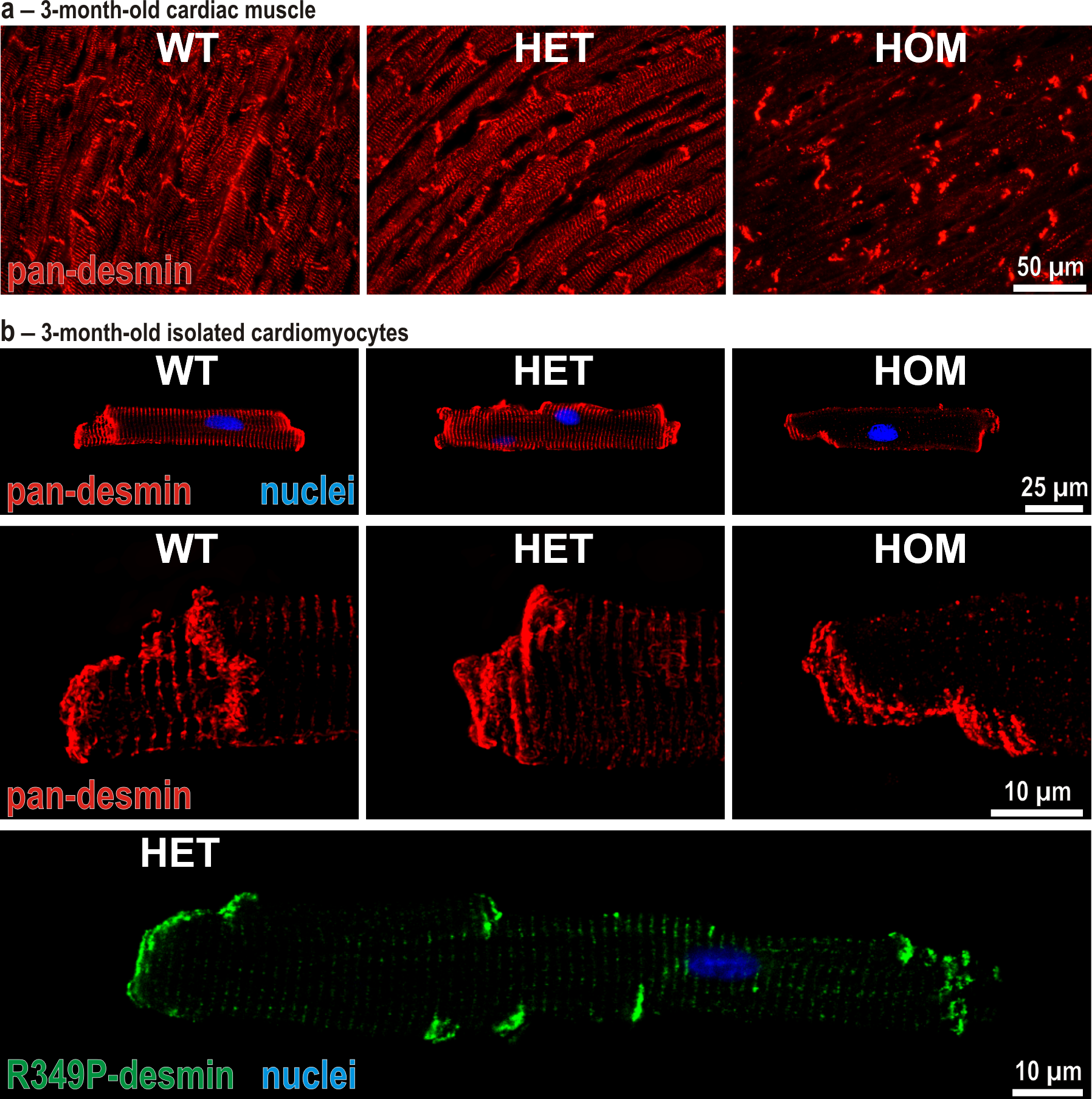

Supplement: Supplementary file 6 — Supplementary material 6 (TIFF 1880 kb) [file 401_2014_1363_MOESM6_ESM.tif]

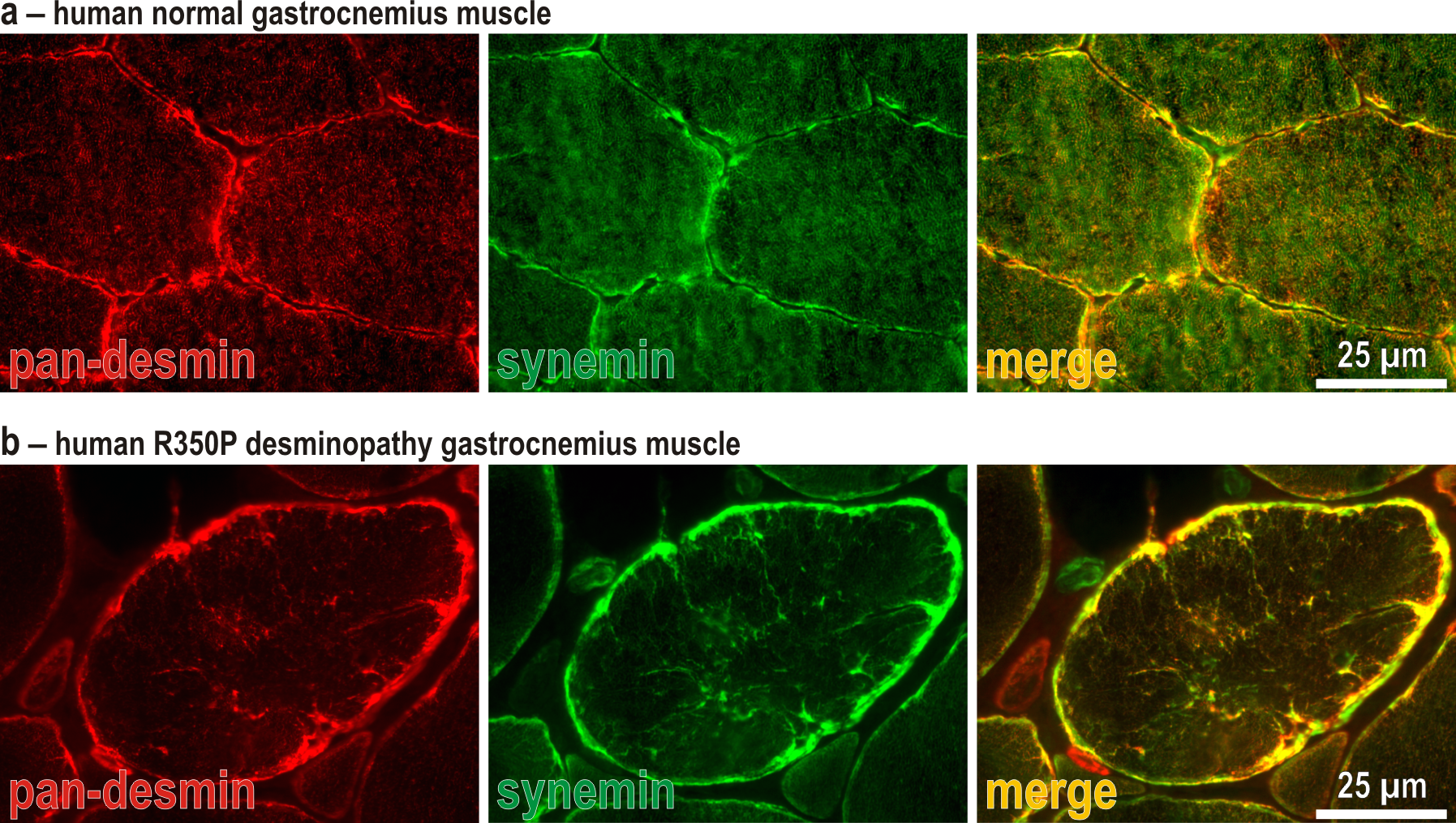

Supplement: Supplementary file 7 — Supplementary material 7 (TIFF 2398 kb) [file 401_2014_1363_MOESM7_ESM.tif]

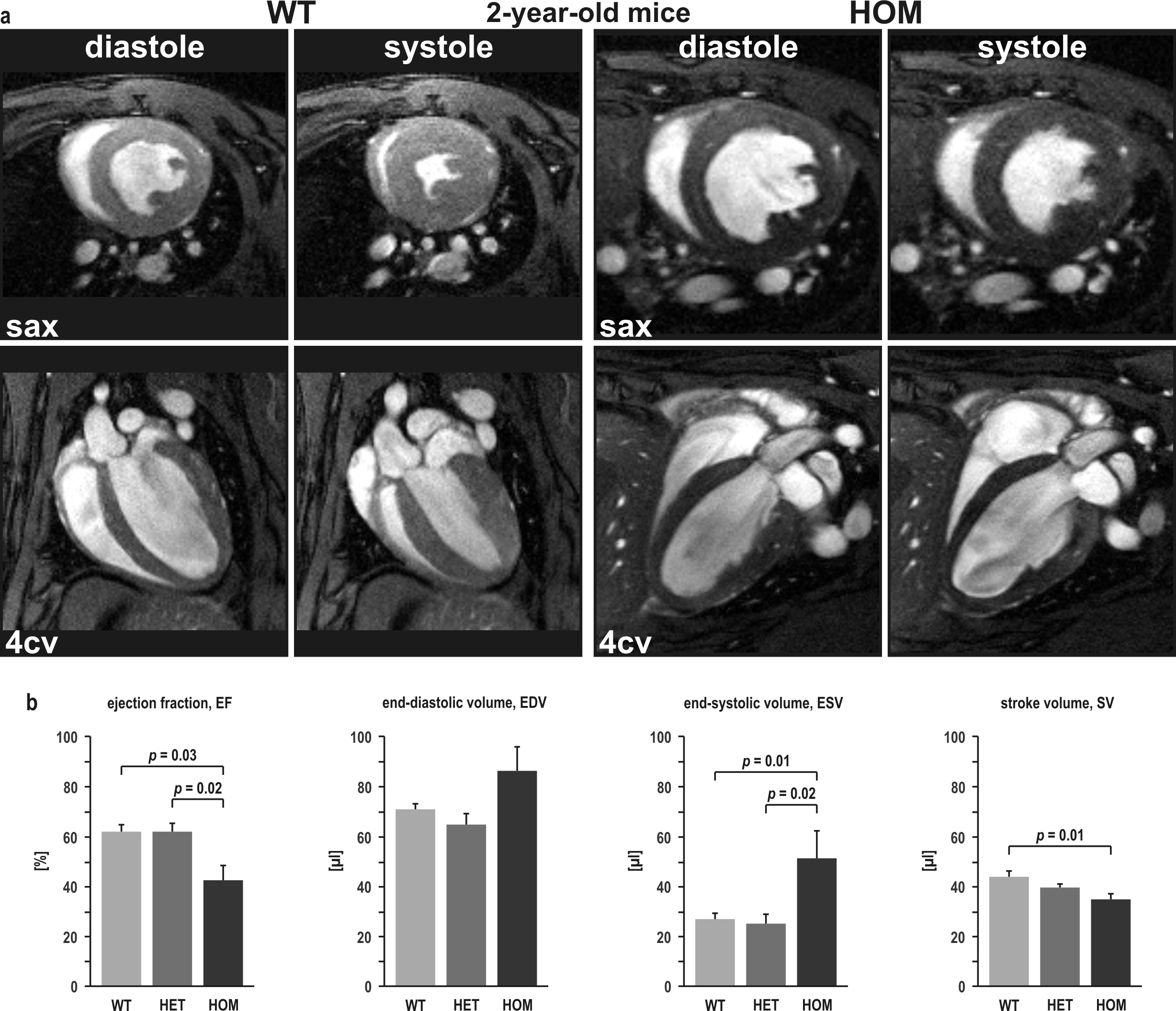

Supplement: Supplementary file 8 — Supplementary material 8 (TIFF 1303 kb) [file 401_2014_1363_MOESM8_ESM.tif]

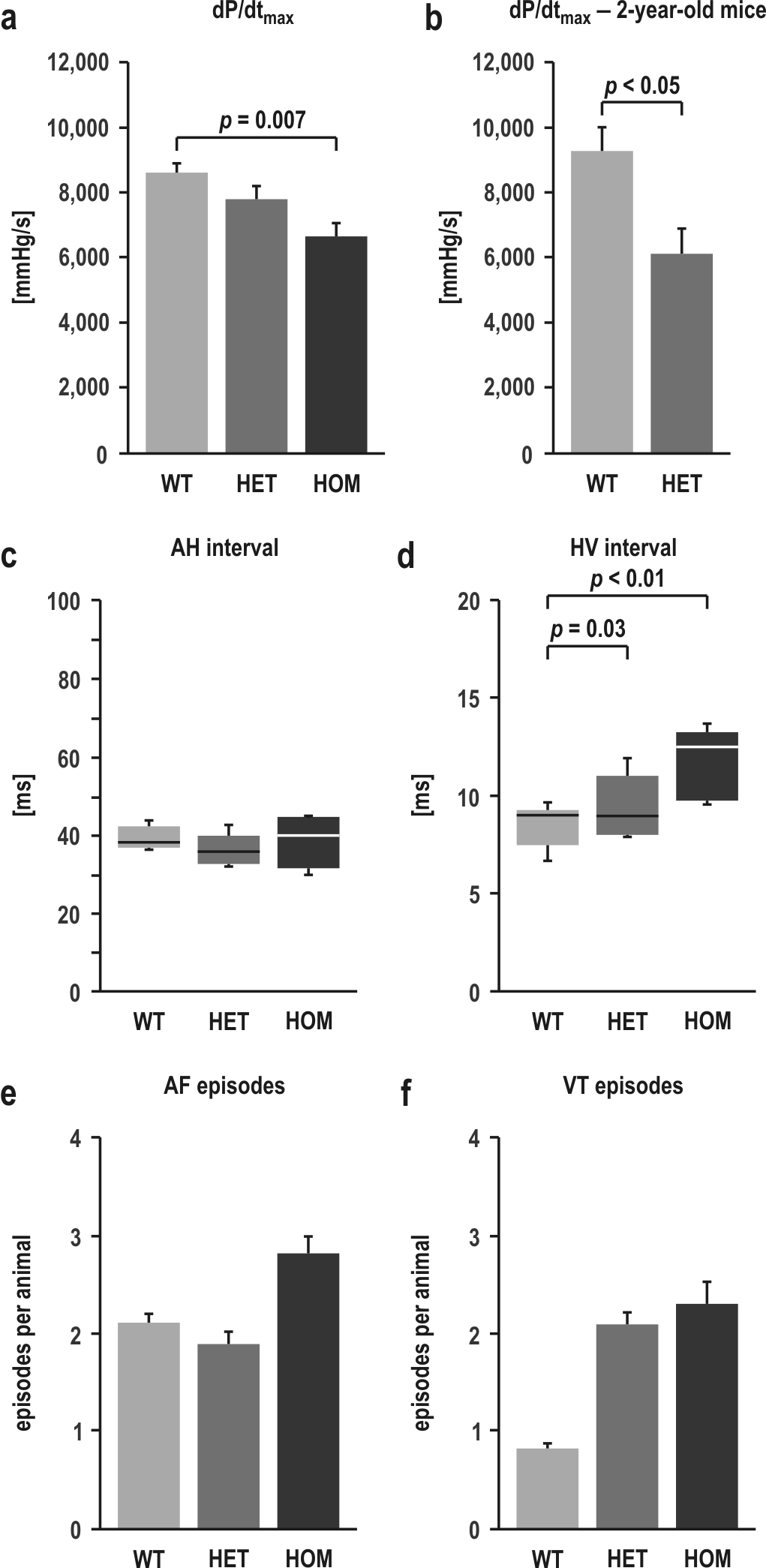

Supplement: Supplementary file 9 — Supplementary material 9 (TIFF 136 kb) [file 401_2014_1363_MOESM9_ESM.tif]

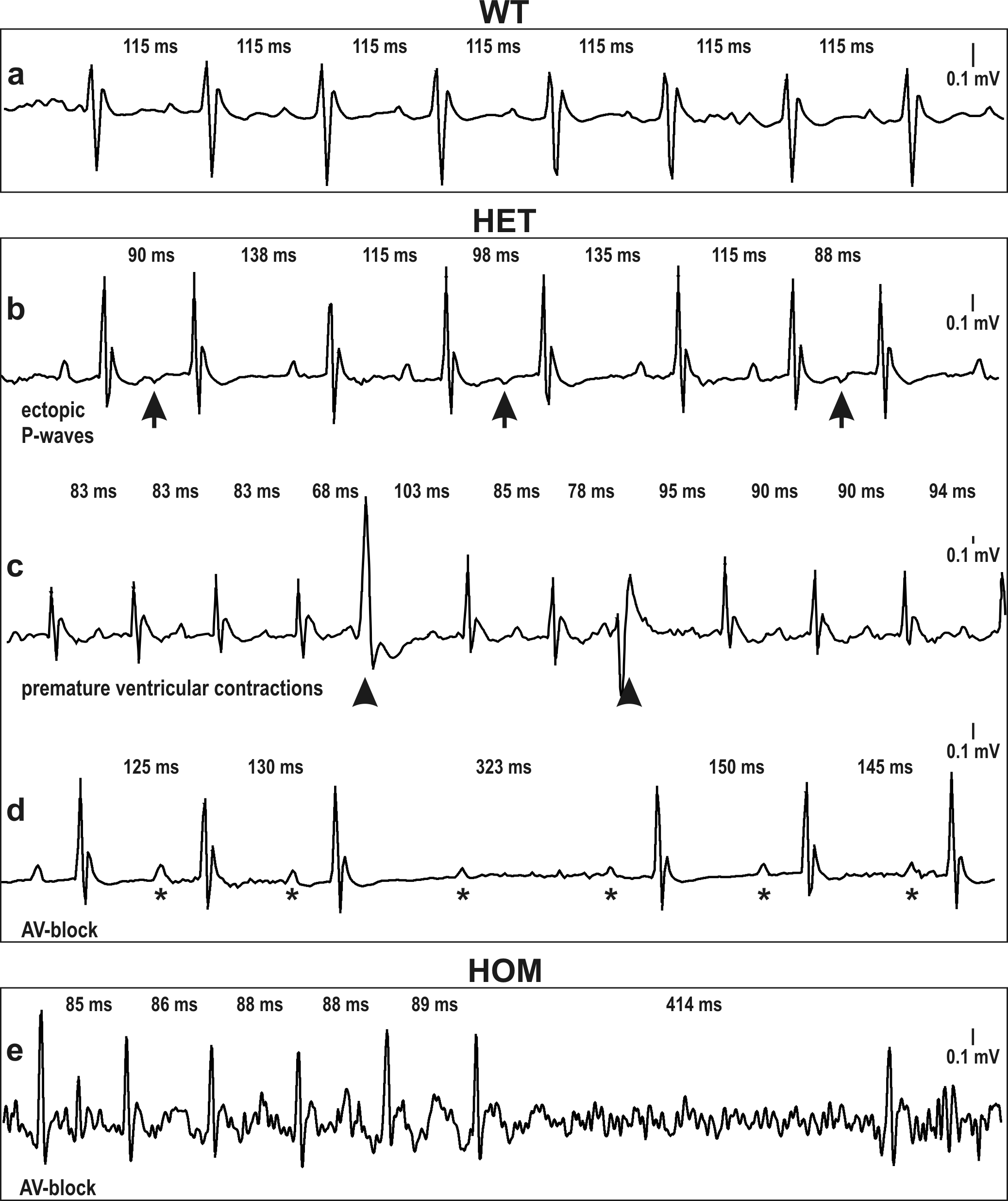

Supplement: Supplementary file 10 — Supplementary material 10 (TIFF 368 kb) [file 401_2014_1363_MOESM10_ESM.tif]

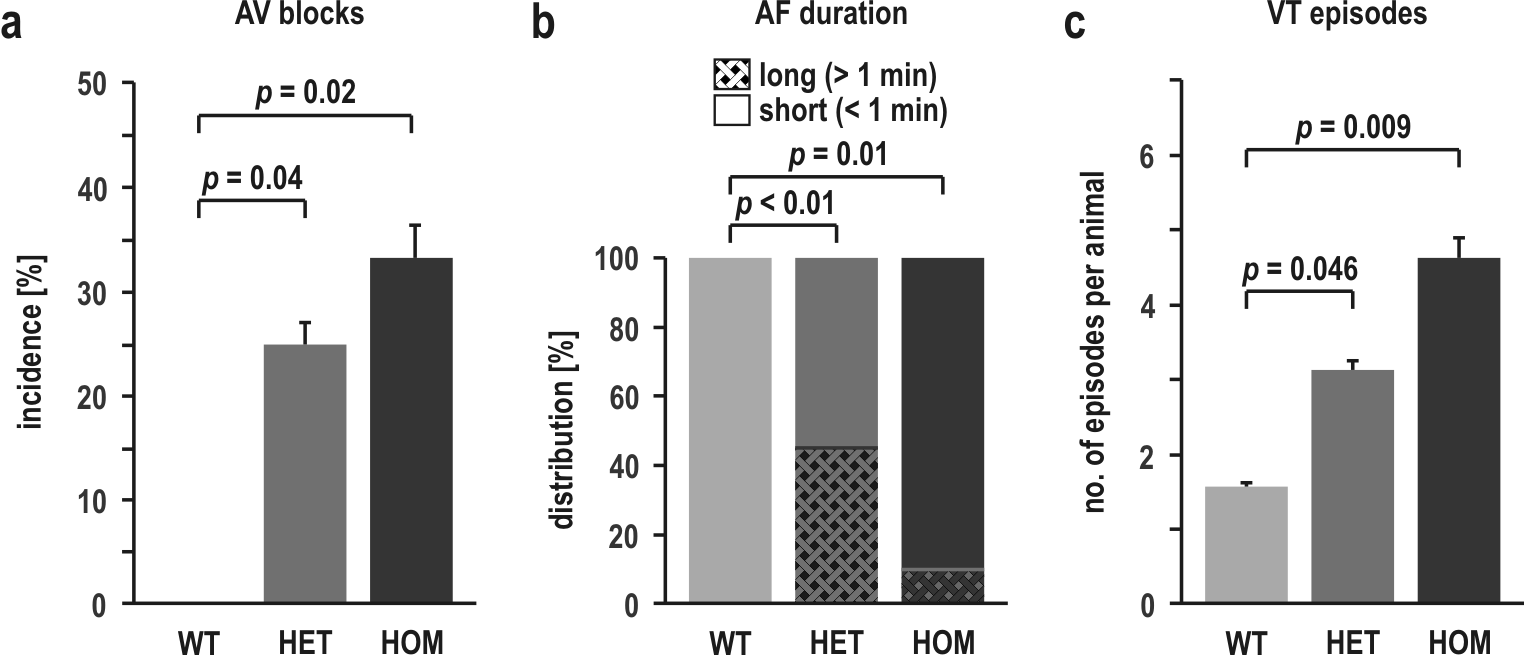

Supplement: Supplementary file 11 — Supplementary material 11 (TIFF 87 kb) [file 401_2014_1363_MOESM11_ESM.tif]
